# Update on immune‐based therapy strategies targeting cancer stem cells

**DOI:** 10.1002/cam4.6520

**Published:** 2023-09-12

**Authors:** Amirhossein Izadpanah, Niloufar Mohammadkhani, Mina Masoudnia, Mahsa Ghasemzad, Arefeh Saeedian, Hamid Mehdizadeh, Mansour Poorebrahim, Marzieh Ebrahimi

**Affiliations:** ^1^ Department of Stem Cells and Developmental Biology, Cell Science Research Center Royan Institute for Stem Cell Biology and Technology, ACECR Tehran Iran; ^2^ Department of Clinical Biochemistry School of Medicine, Shahid Beheshti University of Medical Sciences Tehran Iran; ^3^ Department of Immunology School of Medicine, Shahid Beheshti University of Medical Sciences Tehran Iran; ^4^ Department of Molecular Cell Biology‐Genetics, Faculty of Basic Sciences and Advanced Technologies in Biology University of Science and Culture Tehran Iran; ^5^ Radiation Oncology Research Center Cancer Research Institute, Tehran University of Medical Sciences Tehran Iran; ^6^ Department of Radiation Oncology Cancer Institute, Imam Khomeini Hospital Complex, Tehran University of Medical Sciences Tehran Iran; ^7^ Arnie Charbonneau Cancer Research Institute, University of Calgary Alberta Calgary Canada; ^8^ Department of regenerative medicine Cell Science research Center, Royan Institute for stem cell biology and technology, ACECR Tehran Iran

**Keywords:** cancer stem cells, cell therapy, CSCs biomarker, drug resistance, immunotherapy, targeting CSCs

## Abstract

Accumulating data reveals that tumors possess a specialized subset of cancer cells named cancer stem cells (CSCs), responsible for metastasis and recurrence of malignancies, with various properties such as self‐renewal, heterogenicity, and capacity for drug resistance. Some signaling pathways or processes like Notch, epithelial to mesenchymal transition (EMT), Hedgehog (Hh), and Wnt, as well as CSCs' surface markers such as CD44, CD123, CD133, and epithelial cell adhesion molecule (EpCAM) have pivotal roles in acquiring CSCs properties. Therefore, targeting CSC‐related signaling pathways and surface markers might effectively eradicate tumors and pave the way for cancer survival. Since current treatments such as chemotherapy and radiation therapy cannot eradicate all of the CSCs and tumor relapse may happen following temporary recovery, improving novel and more efficient therapeutic options to combine with current treatments is required. Immunotherapy strategies are the new therapeutic modalities with promising results in targeting CSCs. Here, we review the targeting of CSCs by immunotherapy strategies such as dendritic cell (DC) vaccines, chimeric antigen receptors (CAR)‐engineered immune cells, natural killer‐cell (NK‐cell) therapy, monoclonal antibodies (mAbs), checkpoint inhibitors, and the use of oncolytic viruses (OVs) in pre‐clinical and clinical studies. This review will mainly focus on blood malignancies but also describe solid cancers.

## Graphical abstract

Targeting of CSCs by novel immune‐based therapeutic strategies.

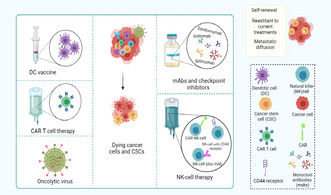

## 1 INTRODUCTION

According to the theory of cancer‐initiating cells (CICs), tumors consist of a vast number of cancer cells alongside a population of tumor cells named CSCs with distinctive properties such as self‐renewal, metastatic diffusion and resistance to conventional cancer therapies which comprise 0.01%–10% of the cells present in the tumor microenvironment (TME).^1^, ^2^, ^3^ Besides the expression of unique CD markers such as CD44, CD24, and CD133, the capacity to express biomarkers like drug‐efflux pumps (e.g., ATP‐binding cassette (ABC)), some enzymes such as aldehyde dehydrogenase (ALDH) and transcription factors (e.g., OCT‐4, SOX‐2) CSCs could be distinguished from the entire cells within the tumor.^4^ This subpopulation was first discovered in acute myeloid leukemia (AML) in 1997.^5^ CSCs have been further identified in various types of solid tumors.^6^ Owing to the specific properties that CSCs demonstrate, like differential ability and capacity to xenograft cancer, quiescence, plasticity, heterogenicity, self‐renewal, accounting for drug resistance, tumor recurrence, immune escaping, and metastasis to other organs, there are a range of predicaments in the way of erasing these cells from the TME.^7^ With concern to the crucial role of CSCs subpopulation in different tumors, in this review, we first elaborated on the CSCs properties and the efficiency of the current cancer therapies including, chemotherapy and radiotherapy. Further, we discussed various immunotherapy approaches for eradicating CSCs.

## 2 CSCs CHARACTERISTICS

### 2.1 CSC signaling pathways

One of the proposed methods for preventing the expansion and eradication of CSCs is targeting the implicated pathways. It is commonly observed that multiple signaling pathways such as Hh, Notch, TGFβ, PI3K/Akt, EGFR, JAK/STAT, and Wnt are dysregulated in CSCs, which confer various properties such as chemoresistance, metastasis, and maintaining their stemness.^8^ The role of the Hh pathway in embryonic development has been highlighted in several solid neoplasms. Depending on the type of tumor, dysregulation of this signaling pathway might cause different clinical outcomes such as chemotherapy resistance in AML cell lines.^9^, ^10^ Three main inhibitors proposed to abort the Hh pathway include antagonists against the transcription factor Smoothened (SMO) (e.g., Vismodegib, Taladegib, Itraconazole), GLI (e.g., ATO, GANT61, Pirfenidone), and Hh ligands (Robotnikinin, HHIP, 5E1).^11^, ^12^, ^13^, ^14^, ^15^ Notch signaling pathway is another conserved pathway of CSCs with implications in immune evasion, metastasis, and angiogenesis. This signaling pathway has been targeted with inhibitors against transcription mediators (IMR‐1, CB‐103, and SAHM1), mAbs against Notch ligands and receptors (e.g., Tarextumab, Enoticumab, Demcizumab), γ‐secretase inhibitors (GSIs) (e.g., MK‐0752, BMS‐906024), and anti‐nicastrin mAbs. Majority of these inhibitors have been entered into phase I or II clinical trials.^16^, ^17^, ^18^, ^19^, ^20^, ^21^ In addition, there are some Notch signaling inhibitors with impressive pre‐clinical outcomes. For instance, GSI PF‐03084014 is a Notch signaling inhibitor that has shown promising pre‐clinical results in the treatment of T‐cell acute lymphoblastic leukemia (T‐ALL) when combined with glucocorticoids.^22^ The Wnt signaling pathway contributes to the embryogenesis and repair system, and its dysregulation is correlated with the initiation and progression of several tumors such as lung, breast, oral, and colorectal tumors.^23^ Application of Vantictumab targeting the Wnt‐related Frizzled receptor family was associated with a reduction in the abundance of CSCs and suppression of tumor growth in xenograft models.^24^ Another inhibitor of the Wnt pathway, CWP232291, with the capacity to facilitate degradation of β‐catenin, demonstrated efficient anti‐cancer activity in mouse models of multiple myeloma (MM).^25^

### 2.2 CSCs surface markers

One of the common approaches for killing CSCs is to target their surface markers. Although the majority of the surface markers expressed on the CSCs are not CSC‐specific, targeting these molecules in combination with other therapies could be effective. CD24, CD44, CD133, CD123, CD47, ALDH, CD34, 5 T4, and EpCAM (ESA) are some examples of the most common surface markers that have been considered for mAbs development.^8^, ^26^ For instance, CD44, a cell adhesion molecule, is one of such important markers that has crucial roles in self‐renewal, tumor proliferation, EMT, niche preparation, apoptosis resistance, and metastasis which can enhance tumor cell stemness through interplaying with extracellular matrix components, growth factors and cytokines.^27^, ^28^ In a recent study, the impact of anti‐CD44 mAb A3D8 on the growth and apoptosis of sphere‐forming cells (SFCs) from the human ovarian cancer cell line SKOV‐3 was assessed. Data indicated that the cell cycle was arrested in the S phase and the expression level of caspase‐3 was up‐regulated, while CDK2, cyclin A, and Bcl‐2 protein expression levels were down‐regulated in the A3D8‐treated cells.^29^ In a pre‐clinical study performed by Jin et al,^30^ anti‐CD44 mAb H90 could effectively hinder the homing of leukemic cells including primitive CD34^+^CD38^−^ SL‐ICs (SCID– leukemia‐initiating cells) to bone marrow and spleen. Furthermore, H90 administration substantially abrogated the transmigration process, abolishing the competence of AML LSCs (leukemic stem cells) to access the bone marrow microenvironment. This phenomenon altered the fate of stem cells in NOD/SCID mouse models with human AML. Thus, targeting CD44 using H90 administration decreased leukemic repopulation and demonstrated CD44 as a key player in the regulation of AML LSCs. AML LSCs interact with a stem cell‐supportive niche to maintain their stem cell characteristics, and these findings suggested a therapeutic approach to target quiescent AML LSCs. CD133 or human prominin‐1, which is a pentaspan membrane glycoprotein, is also highly expressed on the surface of CSCs, and its aberrant expression is correlated with poor prognosis and chemo‐ and radio‐therapy resistance.^29^ In an experiment conducted by Kato et al,^31^ CMab‐43, an anti‐CD133 mAb, was administered in nude mice transplanted with Caco‐2 tumor cells (human colon cancer cell line). Following CMab‐43 administration, the size of the tumor was significantly decreased when compared to the control group treated with a mouse IgG on days 12, 14, and 17. EpCAM (ESA) or CD326 is another surface marker with a high expression in CSCs.^32^, ^33^ It is primarily expressed in simple epithelia, progenitor cells, stem cells from both healthy and malignant origin, and in multiple carcinomas.^34^ EpCAM possesses numerous functions including cell–cell adhesion, proliferation, differentiation, migration, and invasion. However, EpCAM is substantially expressed in carcinoma cells, and due to this fact, the majority of its functions have been primarily identified in malignant cells, and its activity in normal cells is not yet well‐characterized.^35^ EpCAM mediates oncogenic functions in tumor cells. For instance, the EPCAM‐positive cell population demonstrated stem cell characteristics including self‐renewal and pluripotency. They also revealed tumorigenic effects following injection into the NOD‐SCID mice with hepatocellular carcinoma (HCC).^32^ CD47 is a transmembrane immunoglobulin and a receptor for thrombospondin family members that also acts as the signal regulatory protein alpha (SIRPα) ligand mediating several protein–protein interactions.^33^ Phagocytic cells including macrophages and DCs express SIRPα that commences a signaling cascade leading to the phagocytosis prevention (known as the “don't eat me” signal) after engagement with CD47.^36^ Therefore, CD47 overexpression in tumor cells results in the inhibition of phagocytosis by tumor‐associated macrophages (TAM) and is crucial for the survival and proliferation of tumor cells as well as metastasis of hematopoietic malignancies and solid tumors.^36^ Studies have indicated that expression of CD47 is much higher in the AML SCs than their normal counterparts, including hematopoietic stem cells (HSCs) and multipotent progenitor cells.^37^ Table 1 has listed the frequent surface markers that are recruited for the isolation of CSCs from various cancer types.

**TABLE 1:** CSCs biomarkers in different cancer types.

Tumor type	Biomarkers	References
Colorectal cancer	CD133, CD24, CD29, CD44, CD166, EpCAM, Lgr5, ALDH, ESA	38, 39, 40
Gastric cancer	CD133, CD44, CD24, CD166, EpCAM	41, 42, 43, 44
Head and neck cancer	SSEA‐1, CD44, CD133, CD166	45, 46, 47, 48
Melanoma	ABCB5, CD20	49, 50
Pancreatic cancer	CD133, CD44, CD24, ABCG2, ALDH, EpCAM, ESA	41, 51, 52
Lung cancer	CD133, CD44, ABCG2, ALDH, CD87, SP, CD90	41, 50, 53
Liver cancer	CD133, CD44, CD49f, CD90, ALDH, ABCG2, CD24, ESA, CD13, OVC, EpCAM	38, 49
Leukemia (AML)	CD34, CD38, CD123,	52, 54
Prostate cancer	CD133, CD44, α2β1, ABCG2, ALDH, Integrins, CD166, Trop2, CD117	41, 49, 54
Breast cancer	CD133, CD44, CD24, EpCAM, ALDH‐1	49, 52
Ovarian cancer	CD24, CD44, CD117, CD133, ABCG2, EpCAM	^55^

### 2.3 Microenvironment of CSCs

The cross‐talk between the CSCs and the TME is one of the game‐changing factors that could dictate the behavior of these cells and modify the activities of not only the CSCs subpopulation but other cells including immune cells to optimize the environment in favor of tumor cells and promote tumor growth.^56^, ^57^ Targeting suppressive cells in the TME including regulatory T‐cells (T‐reg) and myeloid‐derived suppressor cells (MDSCs) can enhance the functionality of anti‐tumor effector immune cells, as these suppressive cells release inhibitory factors that lead to the suppression and exhaustion of functional immune cells.^58^ Moreover, the acidic pH of TME is a hallmark of malignant tumor cells, which could also have a deterrence effect on activated immune cells. Hypoxia is a feature of tumor cells in TME which induces the expression of hypoxia‐inducible factor‐1 (HIF‐1) regulating the transcription of various angiogenic factors,^59^ and CSC proliferation and self‐renewal.^60^ Hypoxic condition, hereby, induces tumor neovascularization. Angiogenesis is among the most vital properties of TME as the relentless division of tumor cells requires increased blood flow. Hence, crucial nutrients and oxygen are provided to sustain rapid tumor cell proliferation.^59^ Notably, the activities and the network of CSCs and other tumor cells could induce hypoxia and angiogenesis in TME.^61^ Several experiments have focused on the correlation between CSCs activities and angiogenesis. For instance, it is found that higher level of VEGF is produced by CSCs both in normal and hypoxic conditions compared to the non‐CSC populations. The increased levels of VEGF eventually result in new vascular formation.^62^ In a recent study on colorectal cancer (CRC), an anti‐angiogenic substance, Ginsenoside Rg3, was investigated and its suppressor impact on the stemness and growth of CSCs confirmed both in vitro and in vivo. Besides, the mRNA expression level of several genes participating in the angiogenesis including EGF, FGF‐2, PGF, and PIGF, were deceased following treatment with the RG3 that led to the disrupted vascularization of CRC xenograft.^63^ Last but not least, the complication of the metabolism of CSCs that could be altered by the impact of TME has become a topic of interest recently. In a more recent study, Wang and colleagues revealed that downregulation of molecular pathways participating in the Arf1‐mediated lipid metabolism was associated with defects in the mitochondria metabolism, enhanced ER stress, and the release of damage‐associated molecular patterns (DAMPs). These changes recruited DCs involved in the activation of IFN‐γ‐secreting cytotoxic T lymphocytes that eventually resulted in the eradication of CSCs and tumor suppression. More intriguingly, the killing of CSCs led to the induction of a tumor‐specific immune response through imposing CSC‐specific proteins into antigen presentation pathways, which in turn resulted in durable treatment efficacy.^64^

### 2.4 CSCs‐induced drug resistance

**FIGURE 1:**
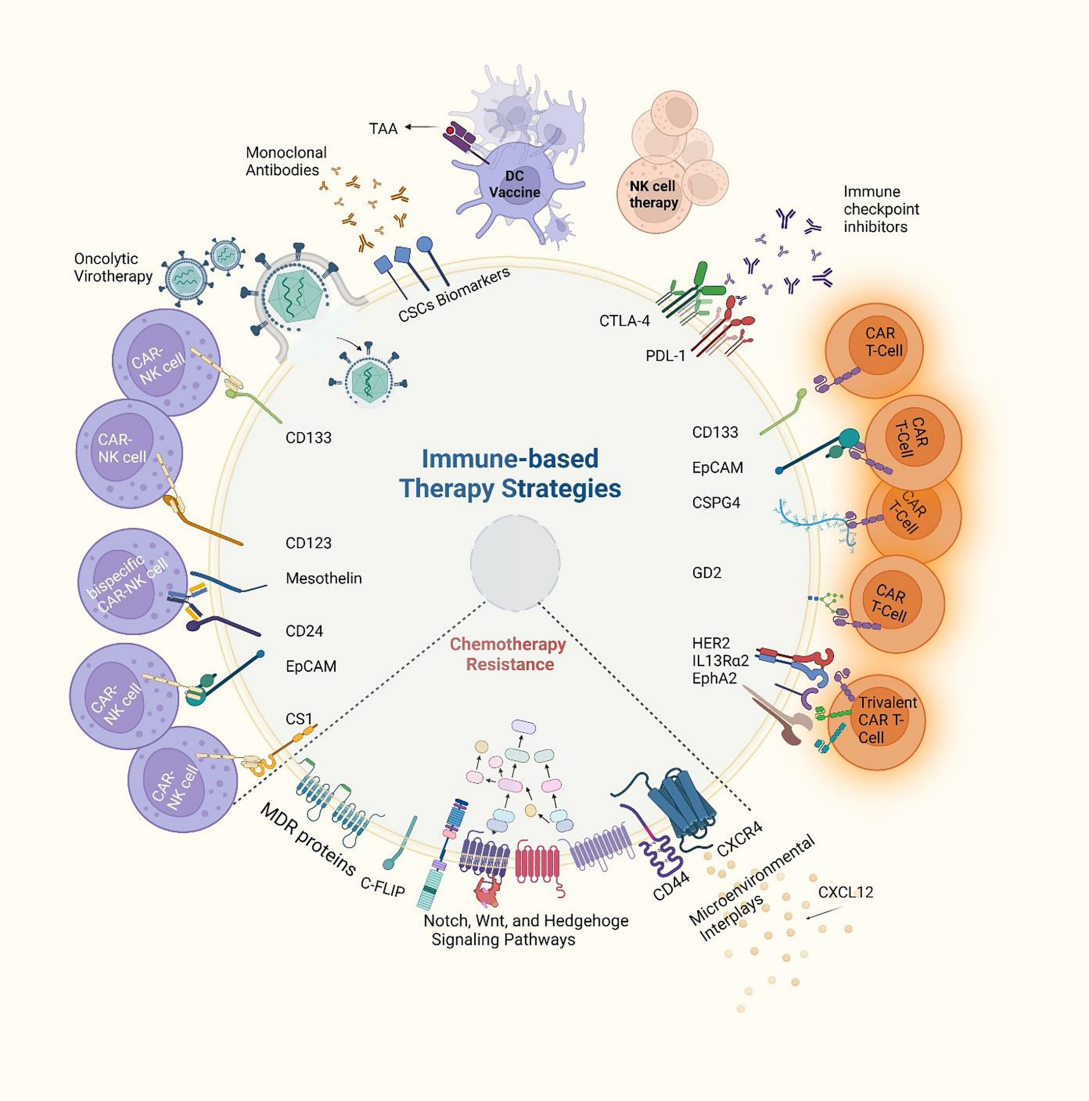
Targeting CSCs. Several strategies have been developed to eradicate CSCs within the tumor, nonetheless, due to specific characteristics of this subpopulation, including overexpression of multidrug resistance (MDR) proteins, multiple signaling pathways as well as interaction with different players of the tumor microenvironment (TME), CSCs exhibit resistance to conventional chemotherapeutics. Novel immune‐based therapy approaches, however, are introduced to efficiently target CSCs. These strategies include: DC vaccines, chimeric antigen receptor (CAR)‐engineered immune cells, natural killer‐cell (NK‐cell) therapy, monoclonal antibodies (mAbs), immune checkpoint inhibitors (ICIs), and oncolytic viruses (OVs).

Drug resistance which is attributed to the presence of CSCs is one of the major issues encountered in cancer therapies. This resistance can stem from low proliferation rate, expression of some transporters like drug‐efflux proteins, autophagy, DNA repair mechanism, and upregulation of ALDH.^65^, ^66^ ALDHs facilitate stem cell maintenance and their proper development and differentiation. They protect the drug‐tolerant cells against elevated levels of reactive oxygen species (ROS)^67^ and also mediate retinoic acid (RA) biosynthesis.^68^ Data report high levels of ALDH activity in various solid tumor types.^69^ Notably, an elevated rate of ALDH activity generally is considered as a negative prognostic indicator.^70^ It is indicated that a pharmacologic decrease of ALDH activity results in toxic levels accumulation of ROS, which consequently eventuates in DNA damage and apoptosis within the drug‐resistant cancer stem cells.^67^ Several experiments have shown that chemotherapy that targets cancer cells with a high proliferation rate, by causing DNA damage or inhibiting the mitotic process, fails to remove CSCs which have a slow rate of proliferation.^71^, ^72^, ^73^ Moreover, the expression of ATP‐binding cassette (ABC) transporters (e.g., P‐gp, MDR1, ABCA1, ABCB1, ABCC11 (MRP8), and ABCG2 (BCRP1)) which take part in transporting conventional chemotherapeutic agents from cytosol to the environment through the energy gained from hydrolysis of ATP, is responsible for the resistance of CSCs to a range of chemotherapeutics and various molecular‐targeted agents leading to lower drug levels in the resistant cells below the amount needed for induction of cell death.^74^, ^75^, ^76^, ^77^ Finally, high expression of apoptotic inhibitors, low levels of Fas and Fas ligand (Fas‐L), and expression of Fas‐associated death domain‐like IL‐1β‐converting enzyme (FLICE)‐inhibitory protein (c‐FLIP) on CSCs could contribute to their resistance to apoptosis^78^, ^79^, ^80^ (Figure 1). C‐FLIP is considered a critical regulator of the death receptor (DR) networks and is a catalytically inactive caspase‐8/‐10 homolog. c‐FLIP protein significantly induces anti‐apoptotic activities through inhibiting cytokine‐ and chemotherapy‐mediated apoptosis, which results in resistance to these agents.^81^ Therefore, targeting drug resistance to increase the sensitivity of CSCs to chemotherapeutic substances or radiotherapy administration could be a promising approach. Cell death regulators such as BCL‐2 have been also targeted in hematopoietic malignancies. However, a proportion of patients treated with venetoclax, a BCL‐2 inhibitor, exhibited minimal residual disease (MRD) which hindered the complete remission rates with the therapeutic strategies based on venetoclax resulting in relapse.^82^ Investigations demonstrated that the retention of leukemic stem cells in the protective niches of bone marrow induced MRD.^83^ C‐X‐C Motif Chemokine Ligand 12 (CXCL12)‐ C‐X‐C Chemokine Receptor Type 4 (CXCR4) signal transduction drives an increase in the cell populations that express high levels of embryonic stem cell core transcription factors (ESC‐TFs: Sox2, Oct4, Nanog) in AML.^84^ Data showed that CD44 was involved in CXCL12‐induced venetoclax resistance of human AML cell lines and AML patient samples.^84^ Yu et al^84^ introduced a novel AML xenograft model in zebrafish and indicated that loss of function of CD44, which physically associates with CXCR4 at the cell membrane upon CXCL12 induction, sensitizes AML cells to the venetoclax through abrogating CXCL12‐mediated survival signaling. This suggests that inhibition of CD44 can be potentially considered to overcome venetoclax‐based therapy resistance in AML.

## 3 CONVENTIONAL STRATEGIES TARGETING CSCs

### 3.1 Chemotherapy

Chemotherapy is one of the cancer treatment strategies. The observed efficacy of the chemotherapy drugs represented that, if a feature is vitally important to tumor biology, its inhibition leads to the prevention of tumor growth. However, the observed clinical responses to these targeted therapies are generally transient and often followed by recurrence. One interpretation, supported by growing empirical evidence, is that each of the main characteristic features is regulated by a set of redundant partial signaling pathways. Thus, tumor cells regulate other pathways following the targeting of a specific pathway. For example, recent pre‐clinical data suggest that hypoxia induction by anti‐angiogenic agents could activate several pathways leading to an aggressive phenotype of tumor through triggering alternative pro‐angiogenic factors and drug resistance.^85^, ^86^, ^87^ Chemotherapy is functional in many cancers at early and advanced stages to eliminate cancer cells and improve survival. However, the effectiveness of conventional chemotherapy drugs on CSCs is limited implying the CSC resistance against various chemotherapy treatments. For instance, imatinib is a BCR‐ABL inhibitor and has shown remarkable efficacy in the recovery and survival of chronic myeloid leukemia (CML) patients.^88^ Nonetheless, in the bone marrow of many patients, BCR‐ABL‐expressing cells fail to be fully eradicated following the imatinib regimen.^89^, ^90^ Besides, in some cases, after achieving complete remission, patients are relapsed following imatinib withdrawal.^90^ Notably, according to the available data, the persistency of CML stem cells resistant to imatinib explains these clinical observations.^90^, ^91^ In the CML‐like mouse model introduced by Oravecz‐Wilson and colleagues, two various CSCs subpopulations including leukemogenic and non‐leukemogenic cells were distinguished through their differences in the expression of specific surface markers. Intriguingly, the leukemogenic cells were scarce and significantly demonstrated higher resistance to imatinib therapy compared to the non‐leukemogenic population.^92^ Accordingly, the properties of CSCs in various cancers play a key role in the progression of cancer and drug resistance. It is of note that the origin of CSCs is not fully understood and might be various in each type of tumor. Furthermore, it is not well known if the population of CSCs is increased upon tumor progression.^93^

### 3.2 Radiotherapy

In addition to chemotherapy, radiotherapy is one of the current cancer treatment options. Radiation therapy destroys cancer cells directly by ionizing radiation that causes DNA damage and indirectly by producing ROS. However, CSCs are considered radioresistant which is associated with an increased ability to repair DNA damage and reduced ROS.^94^, ^95^ Congruently, CSCs in glioma exhibit radiation resistance through induction of DNA damage response,^96^ while in breast cancer, CSCs disclose lower levels of ROS which was described by their anti‐oxidant expression profile leading to radiotherapy resistance.^97^ Radiation is delivered in multiple fractions and doses with improved radiosensitivity by different mechanisms. By fractionations, tumor cells have the opportunity to move into more sensitive phases during cell cycling. Moreover, remaining hypoxic cells will become reoxygenated, which is critical for ionization^97^; although, CSCs are believed to reside in perivascular and hypoxic niches, providing the required condition for them to escape from the radiation effect.^56^ The disadvantage of multi‐fractionated radiotherapy is that the mobilization of CSCs into cell cycling causes the tumor to repopulate, albeit most CSCs are in the G_0_ phase of the cell cycle and proliferate slowly. In conventionally fractionated radiotherapy, the repopulation of tumors is considered the most common reason for therapy failure; the condition in which following the treatment with sublethal doses the re‐progression rate of a tumor is higher than the growth rate of the untreated tumor.^95^, ^97^ Interestingly, local pre‐treatment with radiotherapy could increase the NK‐cell transfer effects.^98^ Moreover, synergistic effects of CSCs‐ targeting T‐cells with radiotherapy on CSCs elimination could be favorable.^99^

## 4 NOVEL IMMUNE‐BASED THERAPEUTIC MODALITIES AGAINST CSCs

With the advances in the understanding of cancer cells and their molecular mechanisms and cell immune cycle that regulate immune response, immunotherapy has been developed for the treatment of hematological malignancies as well as solid tumors. These therapy strategies consist of passive (also known as adaptive) immunotherapy or active immunotherapy. In passive/adaptive approaches, mAbs or tumor‐reactive cells including genetically modified T‐cells, and NK‐cells are used, while in active immunotherapy cells like genetically modified B cells, DCs, and macrophages are applied to elicit an immune response against tumors.^100^ Checkpoint inhibitors and mAbs have provided powerful tools in treating cancer, especially in the advanced and metastatic stages. Cancer immunotherapy is an artificial procedure to stimulate immune system cells to erase cancer cells. Despite the remarkable advances and outcomes of immunotherapy, immunotherapy application against CSCs remains a challenge due to the potential of this subpopulation to escape the immune system by different mechanisms like impaired and downregulation of antigen presentation, modulation of the activity of immune cells, release and expression of immune suppressive factors and receptors, recruiting suppressive cells, and modulation of TME.^101^, ^102^ Subsequently, concerning the urgent necessity to improve the current immunotherapy approaches, the new procedures are focused on CSCs' specific properties in order to beat them. In the following sections, we review the immunotherapy methods introduced for the eradication of CSCs (Figure 1).

### 4.1 Dendritic cell (DC) vaccines

Using the process of antigen presentation by antigen‐presenting cells (APCs) to T‐cells is one of the most studied strategies among adoptive immunotherapy approaches. DCs are among the APCs that elicit both innate and adaptive responses of the host immune system. Via MHC‐II antigen presentation, DCs are capable of activating CD4^+^ T‐cells. CD8^+^ T‐cells are further activated by DCs through a process known as cross‐presentation, in which exogenous antigens are presented on MHC‐I.^103^ Transferring DCs carrying tumor‐associated antigens (TAA) to patients results in the activation of T‐cells, forming immunity against the used antigens and elimination of cancer cells. Applying this approach or cancer vaccination, CSCs could be used as antigens to elicit immune responses.^104^, ^105^ Current advances in CSCs‐targeting vaccination classify DC vaccines into 4 various groups including CSC‐lysates (or inactivated CSC‐based vaccines, CSC‐lysate‐loaded DC vaccines, cytotoxic T‐cell (CTL) vaccines generated with CSC‐lysate‐loaded DC vaccines, plus prophylactic and therapeutic experimental models for combinational strategies.^106^ Experiments on animal models of cancer have illustrated that CSCs lysates could lead to greater responses from T‐cells as it provides the possibility of targeting multiple antigens.^107^ In an experiment using immunocompetent murine tumor models, CSCs‐based vaccination caused the prevention of metastasis of melanoma cells to the lung and inhibition of expansion of the squamous carcinoma.^108^ Additionally, vaccination of mice with CSC lysate‐pulsed DCs significantly increased the lifespan of mice and caused tumor suppression. CSC lysate‐loaded DCs could specifically target CSCs and indicate anti‐tumor potential compared to C57BL/6 mice immunized with the murine melanoma cell line B16F10‐pulsed DCs.^109^ In mouse models of breast cancer, transferring DCs loaded with CSCs lysates could stimulate responses from CD8 and CD45^+^ T‐cells and cause their expansion.^110^ In animal models of D5 melanoma and SCC7 squamous cell cancer, vaccination with ALDH^high^ SCC7 CSC‐DC following surgical excision was effective in reducing tumor recurrence and increasing host survival. In the D5 melanoma, the murine model establishment of ALDH^high^ SCC7 CSC‐DC vaccination was followed by inhibition of tumor growth alongside a reduction in metastasis to the lungs and prolonged survival.^111^ Of note, the combination of the CSC‐DC vaccine and dual blockade of programmed death‐ligand 1(PD1) and cytotoxic T‐lymphocyte‐associated protein (CTLA‐4), in murine models of melanoma, Zheng et al^112^ Proved that triple combination therapy not only could promote the expansion of T‐cell responses against CSCs and inhibit the release of TGF‐β, but it was also more beneficial in the elimination of ALDH CSCs. Of note, the combination of conventional chemotherapy and immunotherapy could be applied to increase the therapy response and also overcome tumor resistance. The administration of dual therapy of CSCs‐DC vaccine and cisplatin against Solid Ehrlich carcinoma in mice was accompanied by increased apoptosis, reduction in tumor growth, and expansion of MDR and Bcl‐2 and was represented as a promising combinational approach.^113^ In a 9L CSC brain tumor model established by Xu et al,^104^ CSCs were recruited as sources of antigens to prime DCs for human GBM vaccination. Data indicated that CSC‐loaded DCs substantially triggered cytotoxic T lymphocytes against CSCs, with tumor‐bearing animals exhibiting a prolonged survival rate. In a more recent experiment on breast cancer, total RNA was gained from the whole breast cancer cell population as well as CSCs, separately. Intriguingly, DCs pulsed with the total RNA of CSCs significantly acted as a better source for activation of effector T‐cells, consequently, resulting in effective apoptosis of breast cancer cells. Nevertheless, there was a report on resistance from CSCs to apoptosis by effector T‐cells due to high expression of PD1 by the CSCs population which in turn eventuates in the higher apoptosis rate of these effector T‐cells.^114^ Although the preclinical studies on DC vaccines for cancer treatment are promising, the better understanding of DC subtypes, approaches to prevail over the immunosuppressive TME, and novel biomarkers recognition are highly required to achieve more efficient DC vaccines.^115^ Several findings from clinical trials studying CSC‐based DC vaccines also indicate significant tumor‐specific immune responses, some of which correlated with the survival benefits for treated cases.^106^, ^116^ Notably, all clinical studies are ongoing in the first two phases, which are short‐term studies with a time span of 10–13 months. Moreover, these trials are mostly aimed at side effects (safety), vaccine dose identification, and the impact of the vaccine on provoking cellular and humoral immune responses.^106^ However, up until now, no clinical trial has been posted to clinical registries. Thus, more high‐quality clinical studies are needed prior to coming to universal conclusions regarding the clinical efficacy of the CSC‐based DC vaccines. Evidently, due to the suboptimal efficacy of CSC‐based DC vaccination, it is consequently suggested to be applied in combination with other therapy strategies including immunotherapies and conventional approaches.^106^ For instance, findings from a randomized phase II clinical trial indicated significant extended survival rates plus elevated CCL22 and IFN‐γ plasma levels in GBM cases following surgical tumor excision and treatment with conventional chemotherapy or radiation therapy in combination with CSC‐DC vaccine.^117^

### 4.2 CAR‐based immune cell therapy

#### 4.2.1 CAR‐T‐cell therapy

Genetically modified immune cells demonstrate remarkable potential for targeting CSC among all the immunotherapy strategies. T‐cells are crucial in cell‐mediated immunity against tumor cells, and engineered T‐cells, including chimeric antigen receptors (CAR) T and T cell receptor (TCR)‐T‐cells, have indicated promising clinical results that exhibit their therapeutic potential in eliminating tumor progression. Currently, this therapy strategy has exhibited more efficiency specifically in the treatment of hematopoietic malignancies. CAR‐T‐cells are the conventional immune cells that were designed and introduced to direct the CD19 molecule on the human leukemia cell.^100^ Cells engaged in this strategy to trigger anti‐tumor responses could be obtained from patients' blood or healthy donors, and following specific ex vivo modification are infused back into the patients while they have achieved the capacity to express specific receptors for the recognition of TAA and subsequently eliminate them.^118^ Beating the need for MHC‐restricted antigen recognition could enable CAR cells to be engineered to target a vast range of antigens. One of the proposed targets for CAR cells could be CSCs as they play a key role in tumor development, and thus, CSCs‐targeting CAR‐T therapy would effectively accelerate the cancer prognosis.^119^ Data from preclinical studies have displayed encouraging results regarding the CD133^+^ CSCs targeting in solid tumors. Accordingly, CAR‐T‐cell therapy is either in monotherapy strategies in glioblastoma^120^ or by applying combinational chemotherapy in ovarian^121^ and gastric cancer stem cells.^122^ In a clinical trial, the efficacy of CD133‐directed CAR‐T‐cells in patients with ALL, AML, breast, brain, liver, colorectal cancer, and pancreatic and ovarian cancers was studied.^123^, ^124^ In 23 patients (14 diagnosed HCC, 7 with pancreatic carcinomas, and 2 with colorectal carcinomas), CD133‐targeting CAR‐T‐cells were administered which later on illustrated the elimination of CD133^+^ cells, alongside managed toxicity and effective disease stability. Interestingly, in all patients, the duration of response ranged from 9 to 63 weeks. Particularly, sorafenib‐resistant hepatocellular carcinoma patients indicated a median progression‐free survival of 7 months.^124^ Recently, the study by Sangsuwannukul et al^125^ demonstrated promising results indicating the efficacy of the fourth‐generation anti‐CD133‐CAR‐T‐cells in eliminating the CD133‐expressing cholangiocarcinoma stem cells dose‐dependently. EpCAM, also known as CD326, ESA, or EGP40 is an adhesion molecule and has critical participation in cell–cell and cell‐to‐cellular matrix interplay. Clinical and preclinical evaluations engaging CAR‐T‐cells directing EpCAM have been conducted^126^, ^127^ and revealed remarkable efficiency in targeting and elimination of EpCAM‐expressing cells in an ovarian cancer cell line (SKOV3)^128^ and xenografts.^129^ Besides, in a colorectal cancer xenograft model, tumor progression was significantly blocked by EpCAM‐CAR‐T‐cell administration, followed by the increase of cytotoxic cytokines, including interferon‐γ (IFN‐γ) and tumor necrosis factor‐alpha (TNF‐α) that were confirmed via in vitro evaluation.^130^ Moreover, Chondroitin Sulfate Proteoglycan 4 (CSPG4), Disialoganglioside (GD2), CD44v6, Interleukin‐13 Receptor α2 (IL13Rα2) as well as CD133 plus CD33 (both of which are leukemic stem cell markers (LSC)) are multiple CSCs markers that are targeted by CAR‐T‐cells in different clinical trials and preclinical studies^131^, ^132^, ^133^ (NCT04097301). Beard et al^131^ reported for the first time that glioblastoma CSCs express CSPG4 on their surface. They generated glioblastoma CSCs from resected human tumors and indicated that anti‐CSPG4 CAR‐ T‐cells have significant capability to recognize and eliminate these cells. The generation of anti‐GD2 CAR‐T‐cells against breast cancer stem‐like cells and administration of them in the xenograft model of TNBC was accompanied by the prevention of local tumor growth and lung metastasis.^133^ Notably, multi‐target CAR‐T‐cells also have been introduced. For example, in primary GBM samples, CAR‐T‐cells engineered to express multiple antigens, including Her2, IL13Rα2, and Ephrin‐A2 (EphA2) confirmed to overcome antigenic heterogeneity and to enhance the therapeutic efficacy in xenograft models.^134^ Although it should be noted that, as much as the generation of CAR immune cells engineered to target specific antigens might seem promising, in practice, severe toxicities have been reported upon injection. Accordingly, targeting multiple antigens probably enhances the challenge of on‐target/off‐tumor toxicity, as the majority of the antigens are expressed on both malignant and healthy cells.^135^, ^136^ As reported, almost 27% of CSC surface markers are also expressed by normal cells.^135^ Advancement of methodologies recognizing tumor‐specific antigens would increase the efficacy of CAR‐based immunotherapies. Furthermore, the development of other engineered T‐cells, including TCR‐engineered T‐cells, TCR‐like CARs, and TCR‐CARs^137^ designed to target CSCs would be of high interest for future investigations of CSCs immunotherapy strategies. Since CSCs are mutated cells and mutated cells commonly present most of these mutations, including neoepitopes, on their surface via MHC class I molecules, the limitation of MHC recognition by CAR‐designed cells might be overcome through the development of TCR‐based CARs, thus, directing CSCs‐specific neoepitopes can substantially reduce off‐tumor toxicities. Other potential strategies that would be applied to increase safety through bypassing off‐tumor toxicity include the modification of CAR affinity, which remarkably contributes to binding and cytotoxicity induction. Accordingly, only high‐density TAAs are recognized by CARs, designed with lower affinity to antigens, while low‐density TAAs in healthy cells are ignored.^22^ Another approach could be the administration of antigen‐specific inhibitory CAR‐T cells. In this strategy, CAR‐T cells express inhibitory receptors against normal antigens along with TAA‐specific receptors. Thus, in the case of interacting with a healthy cell expressing TAA, inhibitory signals hinder cytotoxicity.^127^ Furthermore, employing CAR‐T cells expressing an anti‐CAR would be another potential strategy. In line with this approach, in a more recent experiment, Ruella and colleagues specifically depleted anti‐CD19‐CAR‐T cells using a cellular antidote.^38^ However, we still lack a deeper characterization of these not‐yet well‐studied strategies for the reduction of toxicity following CSCs‐targeting CAR‐T cell therapy.

#### 4.2.2 CAR‐natural killer (NK) cell therapy

NK‐cells are a vital member of innate immunity and exhibit advantages for cancer immunotherapy compared to CAR‐T‐cells. For example, CAR‐NKs kill tumor cells with a lower risk of graft‐versus‐host disease (GvHD) induction. Moreover, CAR‐NK‐cells have a shorter lifespan than T‐cells which leads to a decrease in off‐target toxicities.^138^ CD123 and CD33 are common identification targets for leukemia. Recently, CD33‐CAR‐NK‐92‐cells have entered clinical trials for relapsed/refractory AML (NCT02944162) and case reports investigated the safety and indicated the encouraging tolerability of these AML‐specific CAR‐NK‐cells.^139^ Moreover, in ovarian cancer, targeting CD133 by third‐generation CAR‐NK92 cells significantly prevented tumor progression.^140^ Intriguingly, cisplatin combinational therapy resulted in a higher cell‐killing impact compared to a single treatment strategy, either CAR therapy or chemotherapy.^140^ In another study, researchers developed CAR‐NK‐cells targeting both CD24 and mesothelin which simultaneously directs ovarian CSCs and non‐stem cell tumor cells efficiently.^141^ CAR‐NK92 cells targeting EpCAM on colorectal CSCs have revealed remarkable efficacy in suppressing CRC cell growth.^131^ Notably, a combination of regorafenib with CAR‐NK92 immunotherapy elevated the anti‐cancer effects of therapy in CRC mouse models compared to monotherapy.^142^ Besides, the administration of CAR‐NKs showed effectiveness in the induction of apoptosis in CSCs of MM, as in an experiment on MM patients, the combination therapy of Daratumumab (anti‐CD38) and CAR‐NK‐cells targeting CS1, which is highly expressed in MM CSCs compared to any other cell type, demonstrated potential anti‐tumor effect and inhibited MM relapse through the elimination of MM stem cells.^143^ Figure 1 schematically illustrates novel CAR‐based immune cell therapy against CSCs.

#### 4.2.3 CAR‐macrophage (M) cell therapy

The poor infiltration rate of immune effector cells in the TME is considered an underlying challenge for immunotherapy, specifically for solid tumors. Notably, monocyte‐derived macrophages are the chief players of innate immunity and are the main participants in the TME due to their capacity for penetration into tumor lesions. The enhanced knowledge of TME has introduced novel approaches for applying manipulated macrophages to alleviate immunosuppressive TME, for example, ectopic expression of IL‐21, which stimulates activation of NK/T‐cells at TME.^144^ Data from multiple preclinical studies have confirmed the efficacy of macrophage‐based immunotherapies on tumor suppression, however, still many attempts should be made to optimize the efficacy and safety of CAR‐M in clinical treatment.^145^ Klichinsky et al^146^ utilized primary human macrophages and engineered them to represent sustained pro‐inflammatory phenotypes (M1). Overexpression of pro‐inflammatory chemokines and cytokines by these CAR macrophages equipped them with the accelerated antigen presentation processes and resistance to immunosuppressive cytokines. These CAR‐MS demonstrated significant capacity for alleviation of tumor burden and prolonging overall survival in the xenograft models. Macrophage‐editing‐based immunotherapy strategy, particularly against solid tumors, is a promising direction for future research. Nonetheless, data for CSC‐targeting CAR‐M immunotherapy is not currently available. Thus, this research idea would be a potential avenue in forthcoming research.

### 4.3 NK‐cell therapy

NK‐cells were identified to be naturally cytotoxic to the tumor and damaged cells in 1970. They protect the body against infectious pathogens and tumor cells owing to their intrinsic diverse characteristics and active interplay with adaptive immune cells, including B and T‐cells. NK‐cell populations are categorized into two different subsets of CD56^bright^ and CD16^Dim^ and include 5% to 15% of peripheral blood mononuclear cells (PBMCs).^147^, ^148^ Cytotoxicity effects of allogenic NK‐cells have been evaluated in hematological cancers, while both allogeneic and autologous NK‐cells are efficient against solid tumors. Recently, multiple studies have revealed NK‐cell therapy's capacity to eliminate targeted CSCs.^147^ Experiments on human breast, colon, melanoma, and glioblastoma reveal that IL‐2 and/or IL‐15 activated NK‐cells could identify solid tumor CSCs by involving the receptor‐dependent mechanism, eventually leading to CSC‐elimination in these tumors.^149^, ^150^, ^151^ The Increased sensitivity of CSCs to NK‐cells has been shown in models of colorectal cancer. In this study, allogeneic NK‐cells were recruited to distinguish and eradicate colorectal CSCs. Of note, non‐CSC differentiated tumor cells were less sensitive to NK‐cells, which was correlated with lower expression of NKp30 and NKp44 ligands (in the natural cytotoxicity receptor (NCR) group of activating NK receptors) compared to CSCs. Besides, lower levels of MHC class I are expressed on the CSCs surface, which was indirectly linked to the more efficient targeting of them by NK‐cells.^152^ In our recent experiment on glioblastoma multiform (GBM), the anti‐tumor effect of HSP70/Il‐2‐treated NK‐cells was analyzed and confirmed through both in vitro study and in vivo rat GBM models. Accordingly, NK‐cells could effectively cross the blood–brain barrier following systemic injection and subsequently, target the GMB tumor cells.^153^ Evidence indicates that the combination of drug therapies such as mAbs specific to CSCs markers with NK‐cell therapies could improve the results of cancer treatments and the elimination of CSCs.^6^ For instance, Grossenbacher et al^154^ revealed that co‐incubation of human pancreatic cells with cetuximab could affect against CSCs more efficiently due to its antibody‐dependent‐cell‐mediated cytotoxicity (ADCC) ability. A bispecific fully humanized anti‐CD133 ScFV (single‐chain variable fragment) which bound to CD16 on NK‐cells and CD133 on colorectal cancer CSCs simultaneously, accelerated NK‐cell therapy efficiency.^155^

### 4.4 Monoclonal antibodies (mAbs)

In the last two decades, various mAbs have been approved by FDA for the treatment of cancers such as Rituximab (anti‐CD20), Daratumumab (anti‐CD38) cetuximab (anti‐EGFR), trastuzumab (anti‐HER2) and bevacizumab (anti‐VEGF‐A/anti‐angiogenic) for the treatment of lymphoma, multiple myeloma, epithelial cancer, HER2‐positive breast cancer, respectively.^32^, ^156^, ^157^ MAbs engage the host's immune system for the eradication of targeted cells through triggering humoral and cellular mechanisms, including ADCC, complement‐dependent cytotoxicity (CDC), induction of apoptosis, prevention of receptor‐mediated signal transduction, and activating immune effector cells.^32^ Recent studies in the field of mAbs targeting CSCs have introduced novel approaches. Morita and colleagues introduced CD271 as a CSC biomarker of hypopharyngeal cancer and developed an anti‐CD271 mAb, targeting CD271‐positive cells in xenograft models, eventuated in the decrease of CD271‐expressing CSCs through ADCC mechanism.^158^ Delta‐like ligand 4‐Notch (DLL4‐Notch) signaling plays a key role in protecting chemotherapy‐resistant CSCs. The feasibility of combining standard chemotherapy and anti‐CLLF mAb, demcizumab, and improving the anti‐tumor efficacy was investigated in a phase IB clinical trial.^19^ ROR1 is an oncoembryonic orphan receptor for Wnt5a which is aberrantly expressed in CSCs. Specifically, neoplastic B cells in 95% of CLL patients exhibit ROR1 overexpression. The gene expression signatures of stemness in CLL were prevented following the administration of anti‐ROR1 mAb, cirmtuzumab in vivo. Besides, clinical recruitment of cirmtuzumab effectively suppressed the ROR1 pathway in CLL cases.^159^ MM CSCs aberrantly overexpress ABCG2, known to be the underlying player involved in drug‐efflux and consequent chemotherapy resistance in MM. In vitro and in vivo experiments of Shi and co‐workers indicated that anti‐ABCG2 mAb, conjugated with Epirubicin significantly induced apoptotic signaling pathways in MM CSCs through downregulation of PCNA, Bcl‐2, and CD31 and increased the expression of caspase‐3 and Bax. Various CSCs overexpress the CXCR4, which is correlated with tumorigenicity, angiogenesis, invasion, and drug resistance. Oriuchi et al^160^ developed a radioimmunotherapy strategy using a mAb targeting CXCR4 on AML CSCs in tumor xenografted mice. The strategy demonstrated significant feasibility in targeting and eradicating CSCs of AML. More functionalized mAbs against different targets on CSCs have been categorized in Table 2.

**TABLE 2:** Functionalized mAbs against CSCs.

Name of mAb	Target	In vitro/in vivo	Effects on CSCs	References
Cetuximab	EGFR	In vitro	Killing human pancreatic CSCs by targeting EGFR	^154^
GV5	CD44	In vivo	Elimination of CSCs	^161^
Trastuzumab	HER2	In vitro	Killing of CSCs in breast cancer cells (MCF‐7 and ZR75)	^162^
H4C4	CD44	In vivo	Inhibition of self‐renewal capacity of pancreatic CSCs	^163^
CSL362	CD123	In vitro and in vivo	Lysis of leukemic CSCs	^164^
Adecatumumab (MT201)	EPCAM	In vitro	Killing ovarian cancer CSCs	^165^
H90	CD44	In vivo	Elimination of AML LSC	^166^
P245	CD44	In vivo	Killing of Breast cancer CSCs	^167^
A1MCMMAF	5T4	In vitro and in vivo	Induction of tumor regression Decrease in CSCs population	^168^
Solitomab (MT110)	EpCAM	In vitro and in vivo	Killing of pancreatic and colon CSCs	169, 170
7G3	CD123	In vivo	Killing of AML CSCs	^171^
Fusion of anti‐CD123 ScFV and anti‐CD3 ScFV	CD123 CD3	In vitro	Killing of AML CSCs	^172^
BH6H12	CD47	In vitro and in vivo	Affecting CSCs of brain tumors	32, 173
OMP‐52 M51	Notch 1	In vivo	Killing of breast cancer CSCs	^174^
Demcizumab	DLL4	Phase IB	Metastatic non‐squamous NSCLC	^19^
12C7 and 9B8 from mAb library	–	In vitro	NSCLC	^175^
Figitumumab	IGF	In vitro and in vivo	Reduction in colon cancer cell population and tumor growth	^176^
AVE1642	IGF	In vitro and in vivo	Killing of colon CSCs	^177^

### 4.5 Immune checkpoint inhibitors (ICIs)

Available evidence has revealed that CSCs express more immunosuppressive molecules, including programmed death ligand‐1 (PD‐L1) and T‐lymphocyte antigen‐4 (CTLA‐4), known as immune checkpoints (ICs), compared to their differentiated counterparts.^178^, ^179^, ^180^ ICIs are a promising type of immunotherapy strategy that acts by re‐invigorating the productivity of the host's immune system and suppressing overexpressed receptors or ICs in the microenvironment of cancer cells.^181^, ^182^ CTLA‐4, programmed death receptor‐1 (PD‐1) and PDL‐1 are some of the most well‐known ICs that suppress immune system response which have been already recruited in several clinical studies.^181^ Since CSCs evade the immune system by activating ICs, inhibiting ICs might increase immunity functions and attack CSCs.^183^ CTLA‐4 is a T‐cell receptor that regulates T‐cell activation. Ipilimumab is a known human mAb that blocks CTLA‐4 activation. Clinical studies showed the recruitment of Ipilimumab after chemotherapy could improve the therapeutic efficacy in lung cancer patients.^181^ PD‐1 is an inhibitory T‐cell or B‐cell surface receptor that belongs to the CTLA‐4 family, inflicting the immune system functions once it has bound to its ligand PDL‐1 in various cancers.^181^ Some ICIs such as nivolumab, cemiplimab, and pembrolizumab specifically inhibit PD‐1 and PDL‐1 interactions and enhance T‐cell toxicity against cancer cells.^184^ PDL‐1 is the other checkpoint molecule that causes T‐cell anergy and attenuates immune system functions in different cancers.^181^ It could promote stemness properties, including self‐renewal, tumorigenesis, and drug resistance in CSCs which is linked to the interplay between PDL‐1 and HMGA1 and subsequent activation of PI3K/AKT and MAPK pathways.^185^ Accordingly, in breast cancer, expression of PDL‐1 eventuates in the upregulation of the embryonic stem‐cell markers OCT‐4A, NANOG, and BMI1 which is dependent on the PI3K/AKT signaling pathway.^186^ Furthermore, PDL‐1 expression in gastric CSCs has been related to a higher proliferation rate and chemoresistance.^187^ Zheng et al^112^ examined the CSC‐directing impact of the CSC‐DC vaccine which was combined with a dual suppression of PD‐L1 and CTLA‐4 in an in vivo model for the melanoma tumor. They reported that dual blockade of PD‐L1 and CTLA‐4 significantly augmented the anti‐tumor effect of the CSC‐DC vaccine. Collectively, novel strategies targeting ICs in CSCs or signaling pathways contributed to ICs expression in CSCs would be of high interest for future investigations for improvement of therapeutic efficacy in cancer. Recent clinical trials of immunotherapeutics for CSCs targeting have been listed in Table 3.

**TABLE 3:** Clinical trials of some novel therapeutic modalities targeting CSCs.

Phase	ID number	Approach	Target	Cell‐based therapy	Condition
III	NCT03434379	Checkpoint inhibitors	PDL‐1	–	HCC
–	NCT04977791	Checkpoint inhibitors	PD‐1 and PDL‐1		Non‐small lung cell carcinoma
I/II	NCT02176746	DC vaccine	Colorectal cancer stem cells	–	Colorectal cancer
I/II	NCT00846456	DC vaccine	Tumor stem cells	–	Glioblastoma CSCs
I	NCT01358903	mAbs	CD44	–	Malignant solid tumors
II	NCT03651271	mAbs	CD8	–	Advanced metastatic cancers
I/II	NCT02944162	NK‐cell therapy	CD33	–	Relapsed CD33 AML
I/II	NCT02944162	CAR‐NK‐cell therapy	CD33	NK‐92‐cells	Relapsed/refractory AML
I/II	NCT02541370	CAR‐T‐cell therapy	CD133	Autologous or donor‐derived T‐cells	Liver cancer pancreatic cancer brain tumor
I	NCT03423992	CAR‐T‐cell therapy	CD133, EGFRvIII, IL13RvIII2, Her‐2,EphA2, GD2	Autologous CAR‐T‐cells	Recurrent malignant glioma
I	NCT03563326	CAR‐T‐cell therapy	EpCAM	WCH‐GC‐CAR‐T	Neoplasm, stomach metastases
I	NCT02915445	CAR‐T‐cell therapy	EpCAM	CAR‐T‐cells	Malignant neoplasm of nasopharynx TNM staging distant metastasis (M), Breast cancer recurrent
I	NCT03766126	CAR‐T‐cell therapy	CD123	Autologous CAR‐T‐cells	Relapsed/refractory AML
I	NCT03672851	CAR‐T‐cell therapy	CD123	Autologous CAR‐T‐cells	Relapsed/refractory AML
I	NCT02159495	CAR‐T‐cell therapy	CD123	Autologous/allogeneic CAR‐T‐cells	AML (various) or blastic plasmacytoid dendritic cell neoplasms
I	NCT03114670	CAR‐T‐cell therapy	CD123	Donor‐derived CAR‐T‐cells	Recurred AML after allogeneic hematopoetic stem cell transplantation
I	NCT03126864	CAR‐T‐cell therapy	CD33	Autologous CAR‐T‐cells	Relapsed/refractory AML
I	NCT02799680	CAR‐T‐cell therapy	CD33	Allogeneic CAR‐T‐cells	Relapsed/refractory AML
I/II	NCT04097301	CAR‐T‐cell therapy	CD44v6	Autologous CAR‐T‐cells	AML, MM
I/II	NCT03356782	CAR‐T‐cell therapy	CD133	Autologous CAR‐T‐cells	Sarcoma, osteoid sarcoma, ewing sarcoma
I/II	NCT03013712	CAR‐T‐cell therapy	EpCAM	Autologous CAR‐T‐cells	Colon cancer; esophageal carcinoma; pancreatic, prostate cancer; gastric cancer, hepatic carcinoma
I/II	NCT03556982	CAR‐T‐cell therapy	CD123	Autologous/allogeneic CAR‐T‐cells	Relapsed/refractory AML
I/II	NCT03222674	Multi‐CAR‐T‐cell therapy	CD33, CD38, CD123, CD56, MucI, CLL‐1	Autologous CAR‐T‐cells	Relapsed/refractory AML
I/II	NCT04010877	Multi‐CAR‐T‐cell therapy	CLL‐1, CD33, and/or CD123	Autologous/allogeneic CAR‐T‐cells	AML
I/II	NCT04109482	CAR‐T‐cell therapy	CD123	Autologous CAR‐T‐cells	Relapsed or refractory blastic plasmacytoid dendritic cell neoplasm, acute myeloid leukemia, and high‐risk myelodysplastic syndrome
I/II	NCT01864902	CAR‐T‐cell therapy	CD33	Autologous or donor‐derived T‐cells	Relapsed/refractory AML
II	NCT02725125	CAR‐T‐cell therapy	EpCAM	Autologous CAR‐T‐cells	Relapsed or refractory stomach cancer
II/III	NCT03631576	CAR‐T‐cell therapy	CD123/CLL‐1	CAR‐T‐cells	Relapsed/refractory AML
II	NCT02729493	CAR‐T‐cell therapy	EpCAM	Autologous CAR ‐T‐cells	Relapsed or refractory liver cancer
‐	NCT03473457	Single or double CAR‐T‐cell therapy	CD33, CD38, CD56, CD123, CD117, CD133,CD34, or Mucl	CAR‐T‐cells	Relapsed/refractory AML

### 4.6 Oncolytic virotherapy

Oncolytic viruses (OVs) could reproduce and destroy cells, making them a promising immunotherapy modality in cancer treatment strategies. While unmodified OVs may harm both normal and tumor cells, genetically engineered OVs could distinguish between cancerous and healthy cells, leading to the selective infection and eventual destruction of tumor cells.^188^ Besides, faulty interferon pathways in tumor cells make them more susceptible to infection, especially with some viruses, including vesicular stomatitis virus and myxoma virus.^189^ Evidence suggests multiple viruses possessing oncolytic capacity, that include Poxviridae, Herpesviridae (HSV), Reoviridae (REO), Adenoviridae (AD), Paramyxoviridae, Picornaviridae, and Togaviridae.^189^, ^190^ Talimogene Laherparepvec (TVEC), or Imlygic is one of the FDA‐approved OVs for melanoma treatment, which is a modified HSV.^190^ The other one is Oncorine (H101), a modified AD, which has been applied in the treatment of head and neck cancer.^191^ OVs could be delivered to cancer cells via intratumoral injection or systemically. They function through two direct and indirect pathways. OVs can directly infect and eliminate tumor cells by identifying special biomarkers that are overexpressed on cancer cells like laminin and CD64 or causing immune attack via cytolytic cells indirectly, or both ways together^189^, ^192^ (Figure 1). Current data have shown OVs' ability in targeting CSCs in different types of cancers including brain tumors.^193^ Accordingly, Gp73‐regulated oncolytic AD exhibited toxic effects on hepatic CSCs and induced apoptosis in vitro and in vivo.^194^ In the other study, Jiang et al^195^ examined the anti‐tumor capability of Delta‐24‐RGD, targeted to the abnormal p16INK4/Rb pathway in CSCs of glioblastoma. They reported induced autophagic cell death of CSCs, which was due to the accumulation of Atg5 and LC3‐II protein in cells. In breast cancer patients, CSCs were susceptible to REo, and engineered REO OV was successful in eliminating and lysing both CSCs and non‐CSCs in vitro and in vivo.^196^ Zika virus (ZIKV) has shown a therapeutic oncolytic effect on glioblastoma stem cells (GSCs). It preferentially eliminated patient‐derived GSCs compared with other GBM tumor cells in culture, tumor organoids, and slice cultures.^197^ Recent evidence has indicated that ZIKV targets GSCs and stem‐like cells in medulloblastoma and ependymoma through directing the SOX2‐ integrin αvβ5 pathway.^198^ However, further studies on oncolytic virotherapy require to focus on strategies to improve viral delivery into the tumor, specific targeting of CSCs, as well as enhancing the bioactivity of viruses to survive in the patient's circulation, to reach tumor cells even in distant organs.^199^ Table 4 summarizes various immunotherapy strategies targeting CSCs for a better comparison.

**TABLE 4:** Different immunotherapy approaches for targeting CSCs.

Immunotherapy approach	Advantages	Disadvantages	References
DC vaccines	Cause immunologic memory Targeting a broad range of antigens	Requiring present single antigen Alteration in efficacy after the transition from in vitro to in vivo	200, 201, 202
Engineered T‐cell therapy	Binding with high affinity Production of antigen‐specific, patient‐derived T‐cells Killing cancer cells repeatedly	Short‐term persistence of modified T‐cells in vivo Individual engineering of patient T‐cells is required High risk of autoimmunity	^203^
Nk‐Cell therapy	Easy isolation and expansion ex vivo Recognition of several ligands Antigen non‐specific	Reduced accumulation and activation in the microenvironment of solid tumors	202, 204
mAbs and checkpoint inhibitors	Provide long‐term anti‐tumor response The high survival rate compared to chemotherapy agents	Higher doses are related to a higher risk of treatment‐related death Cause hepatotoxicity or skin‐related troubles Critical neurologic interaction in children Requiring present single antigen	202, 205
Oncolytic viruses	Viral‐mediated cytotoxicity Increased tumor selectivity Enhanced anti‐tumor activity	Efficacy of ADs in vivo is not tested Causing antiviral immunity	^206^

## 5 CHALLENGES AND PERSPECTIVES

Researchers have encountered many obstacles to develop new remedial fields to target CSCs. Perspectives should engage issues, including developing novel delivery methods, preventing toxicity to normal cells, and increased the specificity of targeting CSCs.^38^ Despite the efficacy of immunotherapy approaches on cancer cells and CSCs, the inefficiency of innate and acquired immune systems may happen in some CSCs.^98^ Moreover, the use of specific immunotherapy approaches like receptor/ligand‐based targeting CSCs eliminates some CSCs populations, however, heterogenicity of the present population which has no relevant ligand/receptor could lead to the escape from the antigen‐dependent immunotherapy.^207^ To overcome this problem, some studies suggest that antibodies that are not able to detect surface agents, yet have an elimination effect on CSCs are needed.^33^ Moreover, The main challenge with the CAR‐T‐cell approach is the on/off‐target tumor toxicity which may have toxicity against normal cells.^207^ In addition to them, NK‐cell therapy also is faced with some obstacles including dysfunctionality of autologous NK‐cells and released longevity of NK‐cells in vivo.^98^ In this regard, adaptive delivery of NK‐cells into tumor sites could be more beneficial. Extracellular vesicles (EVs) derived from NK‐cells have the capacity to tolerate the acidic pH of the TME and their nano size may cause encouraging outcomes in visceral tumor treatments.^148^ Furthermore, anti‐cancer drugs or imaging probes‐loaded nanoparticles could pave the way for the treatment and diagnosis of CSCs in a targeted manner.^49^ Yao et al^208^ illustrated that salinomycin‐loaded chitosan‐coated carbon nanotubes could target gastric cancer CSCs and inhibit their self‐renewal potency, migration, and invasion. Since novel treatment paradigms as mentioned in this article could eradicate CSCs, as well as conventional methods' effects on the bulk tumor, combination therapy with immunotherapeutic approaches and conventional treatments may improve the cancer treatment results.^32^, ^33^ Data have shown that a combination of chemotherapy agents with OVs can be a better solution as OVs might overcome the chemoresistance of CSCs, furthermore, chemotherapy drugs may accelerate the cytotoxic activity of OVs.^209^ In light of CSCs targeted treatments, more studies on CSCs characteristics and their related signaling pathways are of high importance. Notably, high throughput sequencing strategy and evaluation of expression patterns of CSCs may help to develop novel drugs targeting CSCs.^210^

## 6 CONCLUSION

CSCs are a subpopulation of cancer cells with self‐renewal feature, responsible for tumor recurrence. Conventional treatments such as chemotherapy and radiotherapy function against the bulk of tumors, but relapse of the tumor may happen in some cases as the CSCs still remain. In this regard, developing novel and effective therapeutic options is an urgent need. The use of immunotherapy methods, including DC vaccines, CAR‐T‐cells, NK‐cells, mAbs, checkpoint inhibitors, and OVs could improve the current cancer treatments' effects and specifically eradicate the CSCs. Like all treatment methods, they have some challenges, mentioned during this review. Of note, more preclinical and clinical studies are required for a better understanding and advancement of these new treatment paradigms.

## AUTHOR CONTRIBUTIONS

**Amirhossein Izadpanah:** Conceptualization (equal); data curation (equal); investigation (equal); methodology (equal); project administration (equal); validation (equal); visualization (equal); writing – original draft (equal); writing – review and editing (equal). **Niloufar Mohammadkhani:** Conceptualization (equal); data curation (equal); investigation (equal); project administration (equal); resources (equal); visualization (equal); writing – original draft (equal); writing – review and editing (equal). **Mina Masoudnia:** Investigation (equal); methodology (equal); visualization (equal); writing – original draft (equal); writing – review and editing (equal). **Mahsa Ghasemzad:** Methodology (equal); project administration (equal); resources (equal); writing – original draft (equal); writing – review and editing (equal). **Arefeh Saeedian:** Project administration (equal); validation (equal); writing – original draft (equal); writing – review and editing (equal). **Hamid Mehdizadeh:** Conceptualization (equal); methodology (equal); project administration (equal); writing – original draft (equal); writing – review and editing (equal). **Mansour Poorebrahim:** Conceptualization (equal); data curation (equal); supervision (equal); validation (equal); writing – original draft (equal). **Marzieh Ebrahimi:** Conceptualization (supporting); data curation (supporting); supervision (lead); validation (lead); visualization (supporting).

## CONFLICT OF INTEREST STATEMENT

All authors declare that they have no conflict of interests.

## AVAILABILITY OF DATA AND MATERIALS

The authors declare that [the/all other] data supporting the findings of this study are available within the article [and its supplementary information files].

## Data Availability

Data sharing is not applicable for this study.

## References

[1] Morrison BJ , Steel JC , Morris JC . Reduction of MHC‐I expression limits T‐lymphocyte‐mediated killing of cancer‐initiating cells. BMC Cancer. 2018;18(1):469. 29699516 10.1186/s12885-018-4389-3 PMC5918869

[2] Munro MJ , Wickremesekera SK , Peng L , Tan ST , Itinteang T . Cancer stem cells in colorectal cancer: a review. J Clin Pathol. 2018;71(2):110‐116. 28942428 10.1136/jclinpath-2017-204739

[3] Parada LF , Dirks PB , Wechsler‐Reya RJ . Brain tumor stem cells remain in play. J Clin Oncol. 2017;35(21):2428‐2431. 28640710 10.1200/JCO.2017.73.9540 PMC5516484

[4] Schulenburg A , Blatt K , Cerny‐Reiterer S , et al. Cancer stem cells in basic science and in translational oncology: can we translate into clinical application? J Hematol Oncol. 2015;8(1):16. 25886184 10.1186/s13045-015-0113-9 PMC4345016

[5] Bennett JM , Catovsky D , Daniel MT , et al. Criteria for the diagnosis of acute leukemia of megakaryocyte lineage (M7). A report of the French‐American‐British cooperative group. Ann Intern Med. 1985;103(3):460‐462. 2411180 10.7326/0003-4819-103-3-460

[6] Dianat‐Moghadam H , Heidarifard M , Jahanban‐Esfahlan R , et al. Cancer stem cells‐emanated therapy resistance: implications for liposomal drug delivery systems. J Control Release. 2018;288:62‐83. 30184466 10.1016/j.jconrel.2018.08.043

[7] Batlle E , Clevers H . Cancer stem cells revisited. Nat Med. 2017;23(10):1124‐1134. 28985214 10.1038/nm.4409

[8] Phi LTH , Sari IN , Yang YG , et al. Cancer stem cells (CSCs) in drug resistance and their therapeutic implications in cancer treatment. Stem Cells Int. 2018;2018:5416923. 29681949 10.1155/2018/5416923 PMC5850899

[9] Aberger F , Hutterer E , Sternberg C , Del Burgo PJ , Hartmann TN . Acute myeloid leukemia–strategies and challenges for targeting oncogenic hedgehog/GLI signaling. Cell Commun Signal. 2017;15(1):8. 28122581 10.1186/s12964-017-0163-4 PMC5267446

[10] Queiroz KC , Ruela‐de‐Sousa RR , Fuhler GM , et al. Hedgehog signaling maintains chemoresistance in myeloid leukemic cells. Oncogene. 2010;29(48):6314‐6322. 20802532 10.1038/onc.2010.375

[11] Bendell J , Andre V , Ho A , et al. Phase I study of LY2940680, a Smo antagonist, in patients with advanced cancer including treatment‐naïve and previously treated basal cell carcinoma LY2940680 demonstrates antitumor activity in BCC. Clin Cancer Res. 2018;24(9):2082‐2091. 29483143 10.1158/1078-0432.CCR-17-0723 PMC6422158

[12] Didiasova M , Singh R , Wilhelm J , et al. Pirfenidone exerts antifibrotic effects through inhibition of GLI transcription factors. FASEB J. 2017;31(5):1916‐1928. 28148565 10.1096/fj.201600892RR

[13] Huang L , Walter V , Hayes DN , Onaitis M . Hedgehog–GLI signaling inhibition suppresses tumor growth in squamous lung cancer. Clin Cancer Res. 2014;20(6):1566‐1575. 24423612 10.1158/1078-0432.CCR-13-2195 PMC4136748

[14] Sekulic A , Migden MR , Oro AE , et al. Efficacy and safety of vismodegib in advanced basal‐cell carcinoma. N Engl J Med. 2012;366(23):2171‐2179. 22670903 10.1056/NEJMoa1113713 PMC5278761

[15] Yang L , Xie G , Fan Q , Xie J . Activation of the hedgehog‐signaling pathway in human cancer and the clinical implications. Oncogene. 2010;29(4):469‐481. 19935712 10.1038/onc.2009.392

[16] Astudillo L , Da Silva TG , Wang Z , et al. The small molecule IMR‐1 inhibits the notch transcriptional activation complex to suppress tumorigenesis inhibitor of mastermind recruitment. Cancer Res. 2016;76(12):3593‐3603. 27197169 10.1158/0008-5472.CAN-16-0061 PMC4911243

[17] Chiorean EG , LoRusso P , Strother RM , et al. A phase I first‐in‐human study of enoticumab (REGN421), a fully human delta‐like ligand 4 (Dll4) monoclonal antibody in patients with advanced solid tumors. Clin Cancer Res. 2015;21(12):2695‐2703. 25724527 10.1158/1078-0432.CCR-14-2797

[18] Hu ZI , Bendell JC , Bullock A , et al. A randomized phase II trial of nab‐paclitaxel and gemcitabine with tarextumab or placebo in patients with untreated metastatic pancreatic cancer. Cancer Med. 2019;8(11):5148‐5157. 31347292 10.1002/cam4.2425 PMC6718621

[19] McKeage MJ , Kotasek D , Markman B , et al. Phase IB trial of the anti‐cancer stem cell DLL4‐binding agent demcizumab with pemetrexed and carboplatin as first‐line treatment of metastatic non‐squamous NSCLC. Target Oncol. 2018;13(1):89‐98. 29188408 10.1007/s11523-017-0543-0

[20] Morgan KM , Fischer BS , Lee FY , et al. Gamma secretase inhibition by BMS‐906024 enhances efficacy of paclitaxel in lung adenocarcinoma. Mol Cancer Ther. 2017;16(12):2759‐2769. 28978720 10.1158/1535-7163.MCT-17-0439 PMC5716926

[21] Piha‐Paul S , Munster P , Hollebecque A , et al. Results of a phase 1 trial combining ridaforolimus and MK‐0752 in patients with advanced solid tumours. Eur J Cancer. 2015;51(14):1865‐1873. 26199039 10.1016/j.ejca.2015.06.115 PMC5693226

[22] Samon JB , Castillo‐Martin M , Hadler M , et al. Preclinical analysis of the g‐secretase inhibitor PF‐03084014 in combination with glucocorticoids in T‐cell acute lymphoblastic leukemia. Mol Cancer Ther. 2012;11:1565‐1575. 22504949 10.1158/1535-7163.MCT-11-0938 PMC3392513

[23] Patel S , Alam A , Pant R , Chattopadhyay S . Wnt signaling and its significance within the tumor microenvironment: novel therapeutic insights. Front Immunol. 2019;10:2872. 31921137 10.3389/fimmu.2019.02872 PMC6927425

[24] Gurney A , Axelrod F , Bond CJ , et al. Wnt pathway inhibition via the targeting of frizzled receptors results in decreased growth and tumorigenicity of human tumors. Proc Natl Acad Sci USA. 2012;109(29):11717‐11722. 22753465 10.1073/pnas.1120068109 PMC3406803

[25] Yoon S‐S , Manasanch EE , Min CK , et al. Novel phase 1a/1b dose‐finding study design of CWP232291 (CWP291) in relapsed or refractory myeloma (MM). J Clin Oncol. 2017;35:TPS8058.

[26] Ghasemi F , Sarabi PZ , Athari SS , Esmaeilzadeh A . Therapeutics strategies against cancer stem cell in breast cancer. Int J Biochem Cell Biol. 2019;109:76‐81. 30772480 10.1016/j.biocel.2019.01.015

[27] Morath I , Hartmann TN , Orian‐Rousseau V . CD44: more than a mere stem cell marker. Int J Biochem Cell Biol. 2016;81(Pt A):166‐173. 27640754 10.1016/j.biocel.2016.09.009

[28] Ghosh SC , Neslihan Alpay S , Klostergaard J . CD44: a validated target for improved delivery of cancer therapeutics. Expert Opin Ther Targets. 2012;16(7):635‐650. 22621669 10.1517/14728222.2012.687374

[29] Du YR , Chen Y , Gao Y , Niu XL , Li YJ , Deng WM . Effects and mechanisms of anti‐CD44 monoclonal antibody A3D8 on proliferation and apoptosis of sphere‐forming cells with stemness from human ovarian cancer. Int J Gynecol Cancer. 2013;23(8):1367‐1375. 24257550 10.1097/IGC.0b013e3182a1d023

[30] Jin L , Hope KJ , Zhai Q , Smadja‐Joffe F , Dick JE . Targeting of CD44 eradicates human acute myeloid leukemic stem cells. Nat Med. 2006;12(10):1167‐1174. 16998484 10.1038/nm1483

[31] Kato Y , Ohishi T , Yamada S , et al. Anti‐CD133 monoclonal antibody CMab‐43 exerts antitumor activity in a mouse xenograft model of colon cancer. Monoclon Antib Immunodiagn Immunother. 2019;38(2):75‐78. 30969150 10.1089/mab.2019.0002

[32] Naujokat CJI . Monoclonal antibodies against human cancer stem cells. Immunotherapy. 2014;6(3):290‐308. 24762074 10.2217/imt.14.4

[33] Yamashita T , Budhu A , Forgues M , Wang XW . Activation of hepatic stem cell marker EpCAM by Wnt‐beta‐catenin signaling in hepatocellular carcinoma. Cancer Res. 2007;67(22):10831‐10839. 18006828 10.1158/0008-5472.CAN-07-0908

[34] Went PT , Lugli A , Meier S , et al. Frequent EpCam protein expression in human carcinomas. Hum Pathol. 2004;35(1):122‐128. 14745734 10.1016/j.humpath.2003.08.026

[35] Dollé L , Theise ND , Schmelzer E , Boulter L , Gires O , van Grunsven LA . EpCAM and the biology of hepatic stem/progenitor cells. Am J Physiol Gastrointest Liver Physiol. 2015;308(4):G233‐G250. 25477371 10.1152/ajpgi.00069.2014 PMC4329473

[36] Chao MP , Weissman IL , Majeti R . The CD47‐SIRPα pathway in cancer immune evasion and potential therapeutic implications. Curr Opin Immunol. 2012;24(2):225‐232. 22310103 10.1016/j.coi.2012.01.010 PMC3319521

[37] Majeti R , Chao MP , Alizadeh AA , et al. CD47 is an adverse prognostic factor and therapeutic antibody target on human acute myeloid leukemia stem cells. Cell. 2009;138(2):286‐299. 19632179 10.1016/j.cell.2009.05.045 PMC2726837

[38] Dragu DL , Necula LG , Bleotu C , Diaconu CC , Chivu‐Economescu M . Therapies targeting cancer stem cells: current trends and future challenges. World J Stem Cells. 2015;7(9):1185‐1201. 26516409 10.4252/wjsc.v7.i9.1185 PMC4620424

[39] Langan RC , Mullinax JE , Raiji MT , et al. Colorectal cancer biomarkers and the potential role of cancer stem cells. J Cancer. 2013;4(3):241‐250. 23459666 10.7150/jca.5832 PMC3584837

[40] Yoo SY , Bang SY , Jeong SN , Kang DH , Heo J . A cancer‐favoring oncolytic vaccinia virus shows enhanced suppression of stem‐cell like colon cancer. Oncotarget. 2016;7(13):16479‐16489. 26918725 10.18632/oncotarget.7660 PMC4941329

[41] Hu Y , Fu L . Targeting cancer stem cells: a new therapy to cure cancer patients. Am J Cancer Res. 2012;2(3):340‐356. 22679565 PMC3365812

[42] Zhang C , Li C , He F , Cai Y , Yang H . Identification of CD44+CD24+ gastric cancer stem cells. J Cancer Res Clin Oncol. 2011;137(11):1679‐1686. 21882047 10.1007/s00432-011-1038-5 PMC11828146

[43] Takaishi S , Okumura T , Tu S , et al. Identification of gastric cancer stem cells using the cell surface marker CD44. Stem Cells. 2009;27(5):1006‐1020. 19415765 10.1002/stem.30 PMC2746367

[44] Nguyen PH , Giraud J , Chambonnier L , et al. Characterization of biomarkers of tumorigenic and chemoresistant cancer stem cells in human gastric carcinoma. Clin Cancer Res. 2017;23(6):1586‐1597. 27620279 10.1158/1078-0432.CCR-15-2157

[45] Son MJ , Woolard K , Nam DH , Lee J , Fine HA . SSEA‐1 is an enrichment marker for tumor‐initiating cells in human glioblastoma. Cell Stem Cell. 2009;4(5):440‐452. 19427293 10.1016/j.stem.2009.03.003 PMC7227614

[46] Prince ME , Sivanandan R , Kaczorowski A , et al. Identification of a subpopulation of cells with cancer stem cell properties in head and neck squamous cell carcinoma. Proc Natl Acad Sci USA. 2007;104(3):973‐978. 17210912 10.1073/pnas.0610117104 PMC1783424

[47] Singh SK , Hawkins C , Clarke ID , et al. Identification of human brain tumour initiating cells. Nature. 2004;432(7015):396‐401. 15549107 10.1038/nature03128

[48] Curtarelli RB , Gonçalves JM , Dos Santos LGP , et al. Expression of cancer stem cell biomarkers in human head and neck carcinomas: a systematic review. Stem Cell Rev Rep. 2018;14(6):769‐784. 30076557 10.1007/s12015-018-9839-4

[49] Chen K , Huang YH , Chen JL . Understanding and targeting cancer stem cells: therapeutic implications and challenges. Acta Pharmacol Sin. 2013;34(6):732‐740. 23685952 10.1038/aps.2013.27 PMC3674516

[50] Chanmee T , Ontong P , Kimata K , Itano N . Key roles of hyaluronan and its CD44 receptor in the stemness and survival of cancer stem cells. Front Oncol. 2015;5:180. 26322272 10.3389/fonc.2015.00180 PMC4530590

[51] Yu F , Yao H , Zhu P , et al. Let‐7 regulates self renewal and tumorigenicity of breast cancer cells. Cell. 2007;131(6):1109‐1123. 18083101 10.1016/j.cell.2007.10.054

[52] Yoshida GJ , Saya H . Therapeutic strategies targeting cancer stem cells. Cancer Sci. 2016;107(1):5‐11. 26362755 10.1111/cas.12817 PMC4724810

[53] Zheng Y , Wang L , Yin L , et al. Lung cancer stem cell markers as therapeutic targets: an update on signaling pathways and therapies. Front Oncol. 2022;12:873994. 35719973 10.3389/fonc.2022.873994 PMC9204354

[54] Collins AT , Berry PA , Hyde C , Stower MJ , Maitland NJ . Prospective identification of tumorigenic prostate cancer stem cells. Cancer Res. 2005;65(23):10946‐10951. 16322242 10.1158/0008-5472.CAN-05-2018

[55] Muinao T , Deka Boruah HP , Pal M . Diagnostic and prognostic biomarkers in ovarian cancer and the potential roles of cancer stem cells – an updated review. Exp Cell Res. 2018;362(1):1‐10. 29079264 10.1016/j.yexcr.2017.10.018

[56] Vlashi E , McBride WH , Pajonk F . Radiation responses of cancer stem cells. J Cell Biochem. 2009;108(2):339‐342. 19623582 10.1002/jcb.22275 PMC2865474

[57] Plaks V , Kong N , Werb Z . The cancer stem cell niche: how essential is the niche in regulating stemness of tumor cells? Cell Stem Cell. 2015;16(3):225‐238. 25748930 10.1016/j.stem.2015.02.015 PMC4355577

[58] Nagarsheth N , Wicha MS , Zou W . Chemokines in the cancer microenvironment and their relevance in cancer immunotherapy. Nat Rev Immunol. 2017;17(9):559‐572. 28555670 10.1038/nri.2017.49 PMC5731833

[59] Taleb M , Mohammadkhani N , Bahreini F , Ovais M , Nie G . Modulation of tumor vasculature network: key strategies. Small Struct. 2022;3(6):2100164.

[60] Heddleston JM , Li Z , Lathia JD , Bao S , Hjelmeland AB , Rich JN . Hypoxia inducible factors in cancer stem cells. Br J Cancer. 2010;102(5):789‐795. 20104230 10.1038/sj.bjc.6605551 PMC2833246

[61] Guo W . Concise review: breast cancer stem cells: regulatory networks, stem cell niches, and disease relevance. Stem Cells Transl Med. 2014;3(8):942‐948. 24904174 10.5966/sctm.2014-0020 PMC4116249

[62] Lizárraga‐Verdugo E , Avendaño‐Félix M , Bermúdez M , Ramos‐Payán R , Pérez‐Plasencia C , Aguilar‐Medina M . Cancer stem cells and its role in angiogenesis and vasculogenic mimicry in gastrointestinal cancers. Front Oncol. 2020;10:413. 32296643 10.3389/fonc.2020.00413 PMC7136521

[63] Tang Y‐C , Zhang Y , Zhou J , et al. Ginsenoside Rg3 targets cancer stem cells and tumor angiogenesis to inhibit colorectal cancer progression in vivo. Int J Oncol. 2018;52(1):127‐138. 29115601 10.3892/ijo.2017.4183 PMC5743384

[64] Wang G , Xu J , Zhao J , et al. Arf1‐mediated lipid metabolism sustains cancer cells and its ablation induces anti‐tumor immune responses in mice. Nat Commun. 2020;11(1):220. 31924786 10.1038/s41467-019-14046-9 PMC6954189

[65] Barbato L , Bocchetti M , Di Biase A , Regad TJC . Cancer stem cells and targeting strategies. Cells. 2019;8(8):926. 31426611 10.3390/cells8080926 PMC6721823

[66] Smith AG , Macleod KF . Autophagy, cancer stem cells and drug resistance. J Pathol. 2019;247(5):708‐718. 30570140 10.1002/path.5222 PMC6668344

[67] Raha D , Wilson TR , Peng J , et al. The cancer stem cell marker aldehyde dehydrogenase is required to maintain a drug‐tolerant tumor cell subpopulation. Cancer Res. 2014;74(13):3579‐3590. 24812274 10.1158/0008-5472.CAN-13-3456

[68] Vassalli G . Aldehyde dehydrogenases: not just markers, but functional regulators of stem cells. Stem Cells Int. 2019;2019:3904645. 30733805 10.1155/2019/3904645 PMC6348814

[69] Dhanyamraju PK , Schell TD , Amin S , Robertson GP . Drug‐tolerant Persister cells in cancer Therapy resistance. Cancer Res. 2022;82(14):2503‐2514. 35584245 10.1158/0008-5472.CAN-21-3844 PMC9296591

[70] Tomita H , Tanaka K , Tanaka T , Hara A . Aldehyde dehydrogenase 1A1 in stem cells and cancer. Oncotarget. 2016;7(10):11018‐11032. 26783961 10.18632/oncotarget.6920 PMC4905455

[71] Moore N , Houghton J , Lyle S . Slow‐cycling therapy‐resistant cancer cells. Stem Cells Dev. 2012;21(10):1822‐1830. 21973238 10.1089/scd.2011.0477 PMC3376467

[72] Ajani JA , Song S , Hochster HS , Steinberg IB . Cancer stem cells: the promise and the potential. Semin Oncol. 2015;42(Suppl 1):S3‐S17. 10.1053/j.seminoncol.2015.01.001 25839664

[73] Vidal S , Rodriguez‐Bravo V , Galsky M , Cordon‐Cardo C , Domingo‐Domenech J . Targeting cancer stem cells to suppress acquired chemotherapy resistance. Oncogene. 2014;33(36):4451‐4463. 24096485 10.1038/onc.2013.411

[74] Dean M . ABC transporters, drug resistance, and cancer stem cells. J Mammary Gland Biol Neoplasia. 2009;14(1):3‐9. 19224345 10.1007/s10911-009-9109-9

[75] Deeley RG , Cole SPC . Substrate recognition and transport by multidrug resistance protein 1 (ABCC1). FEBS Lett. 2006;580(4):1103‐1111. 16387301 10.1016/j.febslet.2005.12.036

[76] Huang B , Fu SJ , Fan WZ , et al. PKCε inhibits isolation and stemness of side population cells via the suppression of ABCB1 transporter and PI3K/Akt, MAPK/ERK signaling in renal cell carcinoma cell line 769P. Transl Androl Urol. 2016;376(1):148‐154. 10.1016/j.canlet.2016.03.041 27037060

[77] Safa AR . Resistance to drugs and cell death in cancer stem cells (CSCs). J Transl Sci. 2020;6(3):341. 35330670 10.15761/jts.1000341 PMC8941648

[78] Ding L , Yuan C , Wei F , et al. Cisplatin restores TRAIL apoptotic pathway in glioblastoma‐derived stem cells through up‐regulation of DR5 and down‐regulation of c‐FLIP. Cancer Invest. 2011;29(8):511‐520. 21877938 10.3109/07357907.2011.605412 PMC3255792

[79] Eisele G , Roth P , Hasenbach K , et al. APO010, a synthetic hexameric CD95 ligand, induces human glioma cell death in vitro and in vivo. Neuro Oncol. 2011;13(2):155‐164. 21183510 10.1093/neuonc/noq176 PMC3064626

[80] Tao J , Qiu B , Zhang D , Wang Y . Expression levels of Fas/Fas‐L mRNA in human brain glioma stem cells. Mol Med Rep. 2012;5(5):1202‐1206. 22344564 10.3892/mmr.2012.791

[81] Safa AR . Drug and apoptosis resistance in cancer stem cells: a puzzle with many pieces. Cancer Drug Resist. 2022;5(4):850‐872. 36627897 10.20517/cdr.2022.20 PMC9771762

[82] Jordan CT . Can we selectively target AML stem cells? Best Pract Res Clin Haematol. 2019;32(4):101100. 31779978 10.1016/j.beha.2019.101100

[83] Konopleva M , Tabe Y , Zeng Z , Andreeff M . Therapeutic targeting of microenvironmental interactions in leukemia: mechanisms and approaches. Drug Resist Updat. 2009;12(4–5):103‐113. 19632887 10.1016/j.drup.2009.06.001 PMC3640296

[84] Yu X , Munoz‐Sagredo L , Streule K , et al. CD44 loss of function sensitizes AML cells to the BCL‐2 inhibitor venetoclax by decreasing CXCL12‐driven survival cues. Blood. 2021;138(12):1067‐1080. 34115113 10.1182/blood.2020006343

[85] Ebos JM , Lee CR , Cruz‐Munoz W , Bjarnason GA , Christensen JG , Kerbel RS . Accelerated metastasis after short‐term treatment with a potent inhibitor of tumor angiogenesis. Cancer Cell. 2009;15(3):232‐239. 19249681 10.1016/j.ccr.2009.01.021 PMC4540346

[86] Pàez‐Ribes M , Allen E , Hudock J , et al. Antiangiogenic therapy elicits malignant progression of tumors to increased local invasion and distant metastasis. Cancer Cell. 2009;15(3):220‐231. 19249680 10.1016/j.ccr.2009.01.027 PMC2874829

[87] Mancuso MR , Davis R , Norberg SM , et al. Rapid vascular regrowth in tumors after reversal of VEGF inhibition. J Clin Invest. 2006;116(10):2610‐2621. 17016557 10.1172/JCI24612 PMC1578604

[88] Druker BJ , Guilhot F , O'Brien SG , et al. Five‐year follow‐up of patients receiving imatinib for chronic myeloid leukemia. N Engl J Med. 2006;355(23):2408‐2417. 17151364 10.1056/NEJMoa062867

[89] Bhatia R , Holtz M , Niu N , et al. Persistence of malignant hematopoietic progenitors in chronic myelogenous leukemia patients in complete cytogenetic remission following imatinib mesylate treatment. Blood. 2003;101(12):4701‐4707. 12576334 10.1182/blood-2002-09-2780

[90] Chu S , McDonald T , Lin A , et al. Persistence of leukemia stem cells in chronic myelogenous leukemia patients in prolonged remission with imatinib treatment. Blood. 2011;118(20):5565‐5572. 21931114 10.1182/blood-2010-12-327437 PMC3217358

[91] Graham SM , Jørgensen HG , Allan E , et al. Primitive, quiescent, Philadelphia‐positive stem cells from patients with chronic myeloid leukemia are insensitive to STI571 in vitro. Blood. 2002;99(1):319‐325. 11756187 10.1182/blood.v99.1.319

[92] Oravecz‐Wilson KI , Philips ST , Yilmaz OH , et al. Persistence of leukemia‐initiating cells in a conditional knockin model of an imatinib‐responsive myeloproliferative disorder. Cancer Cell. 2009;16(2):137‐148. 19647224 10.1016/j.ccr.2009.06.007 PMC2763369

[93] Magee JA , Piskounova E , Morrison SJ . Cancer stem cells: impact, heterogeneity, and uncertainty. Cancer Cell. 2012;21(3):283‐296. 22439924 10.1016/j.ccr.2012.03.003 PMC4504432

[94] Liu Y , Yang M , Luo J , Zhou H . Radiotherapy targeting cancer stem cells “awakens” them to induce tumour relapse and metastasis in oral cancer. Int J Oral Sci. 2020;12(1):19. 32576817 10.1038/s41368-020-00087-0 PMC7311531

[95] Ogawa K , Yoshioka Y , Isohashi F , Seo Y , Yoshida K , Yamazaki H . Radiotherapy targeting cancer stem cells: current views and future perspectives. Anticancer Res. 2013;33(3):747‐754. 23482741

[96] Bao S , Wu Q , McLendon RE , et al. Glioma stem cells promote radioresistance by preferential activation of the DNA damage response. Nature. 2006;444(7120):756‐760. 17051156 10.1038/nature05236

[97] Pajonk F , Vlashi E , McBride WH . Radiation resistance of cancer stem cells: the 4 R's of radiobiology revisited. Stem Cells. 2010;28(4):639‐648. 20135685 10.1002/stem.318 PMC2940232

[98] Luna JI , Grossenbacher SK , Murphy WJ , Canter RJ . Targeting cancer stem cells with natural killer cell immunotherapy. Expert Opin Biol Ther. 2017;17(3):313‐324. 27960589 10.1080/14712598.2017.1271874 PMC5311007

[99] Codd AS , Kanaseki T , Torigo T , Tabi ZJI . Cancer stem cells as targets for immunotherapy. Immunology. 2018;153(3):304‐314. 29150846 10.1111/imm.12866 PMC5795182

[100] Poorebrahim M , Abazari MF , Sadeghi S , et al. Genetically modified immune cells targeting tumor antigens. Pharmacol Ther. 2020;214:107603. 32553789 10.1016/j.pharmthera.2020.107603

[101] Triaca V , Carito V , Fico E , et al. Cancer stem cells‐driven tumor growth and immune escape: the Janus face of neurotrophins. Aging (Albany NY). 2019;11(23):11770‐11792. 31812953 10.18632/aging.102499 PMC6932930

[102] Tsuchiya H , Shiota G . Immune evasion by cancer stem cells. Regen Ther. 2021;17:20‐33. 33778133 10.1016/j.reth.2021.02.006 PMC7966825

[103] Garg AD , Coulie PG , Van den Eynde BJ , Agostinis P . Integrating next‐generation dendritic cell vaccines into the current cancer immunotherapy landscape. Trends Immunol. 2017;38(8):577‐593. 28610825 10.1016/j.it.2017.05.006

[104] Xu Q , Liu G , Yuan X , et al. Antigen‐specific T‐cell response from dendritic cell vaccination using cancer stem‐like cell‐associated antigens. Stem Cells. 2009;27(8):1734‐1740. 19536809 10.1002/stem.102 PMC5854496

[105] Zhou L , Lu L , Wicha MS , et al. Promise of cancer stem cell vaccine. Hum Vaccin Immunother. 2015;11(12):2796‐2799. 26337078 10.1080/21645515.2015.1083661 PMC5054775

[106] Hashemi F , Razmi M , Tajik F , et al. Efficacy of whole cancer stem cell‐based vaccines: a systematic review of preclinical and clinical studies. Stem Cells. 2023;41(3):207‐232. 36573273 10.1093/stmcls/sxac089

[107] Pan Q , Li Q , Liu S , et al. Concise review: targeting cancer stem cells using immunologic approaches. Stem Cells. 2015;33(7):2085‐2092. 25873269 10.1002/stem.2039 PMC4478204

[108] Ning N , Pan Q , Zheng F , et al. Cancer stem cell vaccination confers significant antitumor immunity. Cancer Res. 2012;72(7):1853‐1864. 22473314 10.1158/0008-5472.CAN-11-1400 PMC3320735

[109] Dashti A , Ebrahimi M , Hadjati J , Memarnejadian A , Moazzeni SM . Dendritic cell based immunotherapy using tumor stem cells mediates potent antitumor immune responses. Cancer Lett. 2016;374(1):175‐185. 26803056 10.1016/j.canlet.2016.01.021

[110] Van Phuc P , Hou CJ , Nguyet NTM , et al. Effects of breast cancer stem cell extract primed dendritic cell transplantation on breast cancer tumor murine models. Annu Res Rev Biol. 2011;1:1‐13.

[111] Hu Y , Lu L , Xia Y , et al. Therapeutic efficacy of cancer stem cell vaccines in the adjuvant setting. Cancer Res. 2016;76(16):4661‐4672. 27325649 10.1158/0008-5472.CAN-15-2664 PMC4987233

[112] Zheng F , Dang J , Zhang H , et al. Cancer stem cell vaccination with PD‐L1 and CTLA‐4 blockades enhances the eradication of melanoma stem cells in a mouse tumor model. J Immunother. 2018;41(8):361‐368. 30063587 10.1097/CJI.0000000000000242 PMC6128768

[113] El‐Ashmawy N , Salem M , Khedr E , El‐Zamarany E , Ibrahim AO . Dual‐targeted therapeutic strategy combining CSC–DC‐based vaccine and cisplatin overcomes chemo‐resistance in experimental mice model. Clin Transl Oncol. 2020;22(7):1155‐1165. 31748959 10.1007/s12094-019-02242-4

[114] Sumransub N , Jirapongwattana N , Jamjuntra P , et al. Breast cancer stem cell RNA‐pulsed dendritic cells enhance tumor cell killing by effector T cells. Oncol Lett. 2020;19(3):2422‐2430. 32194742 10.3892/ol.2020.11338 PMC7038997

[115] Najafi S , Mortezaee K . Advances in dendritic cell vaccination therapy of cancer. Biomed Pharmacother. 2023;164:114954. 37257227 10.1016/j.biopha.2023.114954

[116] Sheikh NA , Petrylak D , Kantoff PW , et al. Sipuleucel‐T immune parameters correlate with survival: an analysis of the randomized phase 3 clinical trials in men with castration‐resistant prostate cancer. Cancer Immunol Immunother. 2013;62(1):137‐147. 22865266 10.1007/s00262-012-1317-2 PMC3541926

[117] Yao Y , Luo F , Tang C , et al. Molecular subgroups and B7‐H4 expression levels predict responses to dendritic cell vaccines in glioblastoma: an exploratory randomized phase II clinical trial. Cancer Immunol Immunother. 2018;67(11):1777‐1788. 30159779 10.1007/s00262-018-2232-y PMC11028057

[118] Poorebrahim M , Mohammadkhani N , Foad M , et al. CAR‐T cells in brain tumors and autoimmune diseases–from basics to the clinic. Front Clin Drug Res. 2021;9:65.

[119] Walcher L , Kistenmacher AK , Suo H , et al. Cancer stem cells‐origins and biomarkers: perspectives for targeted personalized therapies. Front Immunol. 2020;11:1280. 32849491 10.3389/fimmu.2020.01280 PMC7426526

[120] Zhu X , Prasad S , Gaedicke S , Hettich M , Firat E , Niedermann G . Patient‐derived glioblastoma stem cells are killed by CD133‐specific CAR T cells but induce the T cell aging marker CD57. Oncotarget. 2015;6(1):171‐184. 25426558 10.18632/oncotarget.2767 PMC4381586

[121] Hu B , Zou Y , Zhang L , et al. Nucleofection with plasmid DNA for CRISPR/Cas9‐mediated inactivation of programmed cell death protein 1 in CD133‐specific CAR T cells. Hum Gene Ther. 2019;30(4):446‐458. 29706119 10.1089/hum.2017.234

[122] Han Y , Sun B , Cai H , Xuan Y . Simultaneously target of normal and stem cells‐like gastric cancer cells via cisplatin and anti‐CD133 CAR‐T combination therapy. Cancer Immunol Immunother. 2021;70(10):2795‐2803. 33635343 10.1007/s00262-021-02891-x PMC10991976

[123] Dai H , Tong C , Shi D , et al. Efficacy and biomarker analysis of CD133‐directed CAR T cells in advanced hepatocellular carcinoma: a single‐arm, open‐label, phase II trial. Onco Targets Ther. 2020;9(1):1846926. 10.1080/2162402X.2020.1846926 PMC7714531 33312759

[124] Wang Y , Chen M , Wu Z , et al. CD133‐directed CAR T cells for advanced metastasis malignancies: a phase I trial. Onco Targets Ther. 2018;7(7):e1440169. 10.1080/2162402X.2018.1440169 PMC5993480 29900044

[125] Sangsuwannukul T , Supimon K , Sujjitjoon J , et al. Anti‐tumour effect of the fourth‐generation chimeric antigen receptor T cells targeting CD133 against cholangiocarcinoma cells. Int Immunopharmacol. 2020;89(Pt B):107069. 33242709 10.1016/j.intimp.2020.107069

[126] Deng Z , Wu Y , Ma W , Zhang S , Zhang Y‐Q . Adoptive T‐cell therapy of prostate cancer targeting the cancer stem cell antigen EpCAM. BMC Immunol. 2015;16(1):1. 25636521 10.1186/s12865-014-0064-x PMC4318439

[127] Zhou Y , Wen P , Li M , Li Y , Li XA . Construction of chimeric antigen receptor‐modified T cells targeting EpCAM and assessment of their anti‐tumor effect on cancer cells. Mol Med Rep. 2019;20(3):2355‐2364. 31322180 10.3892/mmr.2019.10460

[128] Fu J , Shang Y , Qian Z , et al. Chimeric Antigen receptor‐T (CAR‐T) cells targeting Epithelial cell adhesion molecule (EpCAM) can inhibit tumor growth in ovarian cancer mouse model. J Vet Med Sci. 2021;83(2):241‐247. 33328392 10.1292/jvms.20-0455 PMC7972873

[129] Ang WX , Li Z , Chi Z , et al. Intraperitoneal immunotherapy with T cells stably and transiently expressing anti‐EpCAM CAR in xenograft models of peritoneal carcinomatosis. Oncotarget. 2017;8(8):13545‐13559. 28088790 10.18632/oncotarget.14592 PMC5355119

[130] Zhang BL , Li D , Gong YL , et al. Preclinical evaluation of chimeric antigen receptor‐modified T cells specific to epithelial cell adhesion molecule for treating colorectal cancer. Hum Gene Ther. 2019;30(4):402‐412. 30693795 10.1089/hum.2018.229

[131] Beard RE , Zheng Z , Lagisetty KH , et al. Multiple chimeric antigen receptors successfully target chondroitin sulfate proteoglycan 4 in several different cancer histologies and cancer stem cells. J Immunother Cancer. 2014;2(1):25. 25197555 10.1186/2051-1426-2-25 PMC4155770

[132] Brown CE , Starr R , Aguilar B , et al. Stem‐like tumor‐initiating cells isolated from IL13Rα2 expressing gliomas are targeted and killed by IL13‐zetakine–redirected T cells. Clin Cancer Res. 2012;18(8):2199‐2209. 22407828 10.1158/1078-0432.CCR-11-1669 PMC3578382

[133] Seitz CM , Schroeder S , Knopf P , et al. GD2‐targeted chimeric antigen receptor T cells prevent metastasis formation by elimination of breast cancer stem‐like cells. Onco Targets Ther. 2020;9(1):1683345. 10.1080/2162402X.2019.1683345 PMC6959445 32002293

[134] Bielamowicz K , Fousek K , Byrd TT , et al. Trivalent CAR T cells overcome interpatient antigenic variability in glioblastoma. Neuro Oncol. 2018;20(4):506‐518. 29016929 10.1093/neuonc/nox182 PMC5909636

[135] Kim WT , Ryu CJ . Cancer stem cell surface markers on normal stem cells. BMB Rep. 2017;50(6):285‐298. 28270302 10.5483/BMBRep.2017.50.6.039 PMC5498139

[136] Guedan S , Calderon H , Posey AD Jr , Maus MV . Engineering and design of chimeric antigen receptors. Mol Ther Methods Clin Dev. 2019;12:145‐156. 30666307 10.1016/j.omtm.2018.12.009 PMC6330382

[137] Poorebrahim M , Mohammadkhani N , Mahmoudi R , Gholizadeh M , Fakhr E , Cid‐Arregui A . TCR‐like CARs and TCR‐CARs targeting neoepitopes: an emerging potential. Cancer Gene Ther. 2021;28(6):581‐589. 33654227 10.1038/s41417-021-00307-7 PMC8203496

[138] Yilmaz A , Cui H , Caligiuri MA , Yu J . Chimeric antigen receptor‐engineered natural killer cells for cancer immunotherapy. Stem Cell Rev Rep. 2020;13(1):1‐22. 10.1186/s13045-020-00998-9 PMC7720606 33287875

[139] Tang X , Yang L , Li Z , et al. First‐in‐man clinical trial of CAR NK‐92 cells: safety test of CD33‐CAR NK‐92 cells in patients with relapsed and refractory acute myeloid leukemia. Am J Cancer Res. 2018;8(6):1083‐1089. 30034945 PMC6048396

[140] Klapdor R , Wang S , Hacker U , et al. Improved killing of ovarian cancer stem cells by combining a novel chimeric antigen receptor‐based immunotherapy and chemotherapy. Hum Gene Ther. 2017;28(10):886‐896. 28836469 10.1089/hum.2017.168

[141] Klapdor R , Wang S , Morgan M , et al. Characterization of a novel third‐generation anti‐CD24‐CAR against ovarian cancer. Int J Mol Sci. 2019;20(3):660. 30717444 10.3390/ijms20030660 PMC6387114

[142] Zhang Q , Zhang H , Ding J , et al. Combination therapy with EpCAM‐CAR‐NK‐92 cells and regorafenib against human colorectal cancer models. J Immunol Res. 2018;2018:4263520. 30410941 10.1155/2018/4263520 PMC6205314

[143] Zhang Y , Chen L , Wang Y , et al. Combination therapy with daratumumab and CAR‐NK targeting CS1 for multiple myeloma. Blood. 2016;128(22):1342.

[144] Moyes KW , Lieberman NA , Kreuser SA , et al. Genetically engineered macrophages: a potential platform for cancer immunotherapy. Hum Gene Ther. 2017;28(2):200‐215. 27758144 10.1089/hum.2016.060

[145] Chen Y , Yu Z , Tan X , et al. CAR‐macrophage: a new immunotherapy candidate against solid tumors. Biomed Pharmacother. 2021;139:111605. 33901872 10.1016/j.biopha.2021.111605

[146] Klichinsky M , Ruella M , Shestova O , et al. Human chimeric antigen receptor macrophages for cancer immunotherapy. Nat Biotechnol. 2020;38(8):947‐953. 32361713 10.1038/s41587-020-0462-y PMC7883632

[147] Oh S , Lee J‐H , Kwack K , Choi S‐W . Natural killer cell therapy: a new treatment paradigm for solid tumors. Cancers (Basel). 2019;11(10):1534. 31614472 10.3390/cancers11101534 PMC6826624

[148] Hu W , Wang G , Huang D , Sui M , Xu Y . Cancer immunotherapy based on natural killer cells: current progress and new opportunities. Front Immunol. 2019;10:1205. 31214177 10.3389/fimmu.2019.01205 PMC6554437

[149] Castriconi R , Daga A , Dondero A , et al. NK cells recognize and kill human glioblastoma cells with stem cell‐like properties. J Immunol. 2009;182(6):3530‐3539. 19265131 10.4049/jimmunol.0802845

[150] Yin T , Wang G , He S , Liu Q , Sun J , Wang Y . Human cancer cells with stem cell‐like phenotype exhibit enhanced sensitivity to the cytotoxicity of IL‐2 and IL‐15 activated natural killer cells. Cell Immunol. 2016;300:41‐45. 26677760 10.1016/j.cellimm.2015.11.009

[151] Ames E , Canter RJ , Grossenbacher SK , et al. NK cells preferentially target tumor cells with a cancer stem cell phenotype. J Immunol. 2015;195(8):4010‐4019. 26363055 10.4049/jimmunol.1500447 PMC4781667

[152] Tallerico R , Todaro M , Di Franco S , et al. Human NK cells selective targeting of colon cancer–initiating cells: a role for natural cytotoxicity receptors and MHC class I molecules. J Immunol. 2013;190(5):2381‐2390. 23345327 10.4049/jimmunol.1201542

[153] Sharifzad F , Mardpour S , Mardpour S , et al. HSP70/IL‐2 treated NK cells effectively cross the blood brain barrier and target tumor cells in a rat model of induced glioblastoma multiforme (GBM). Int J Mol Sci. 2020;21(7):2263. 32218162 10.3390/ijms21072263 PMC7178276

[154] Grossenbacher SK , Ames E , Mac S , et al. Enhanced natural killer cell targeting of cancer stem cells using Cetuximab. J Immunother Cancer. 2014;2(3):P13.

[155] Schmohl J , Gleason M , Dougherty P , Miller JS , Vallera DA . Heterodimeric bispecific single chain variable fragments (scFv) killer engagers (BiKEs) enhance NK‐cell activity against CD133+ colorectal cancer cells. Target. Oncologia. 2016;11(3):353‐361. 10.1007/s11523-015-0391-8 PMC4873478 26566946

[156] Kwiatkowska‐Borowczyk EP , Gąbka‐Buszek A , Jankowski J , Mackiewicz A . Immunotargeting of cancer stem cells. Cancers (Basel). 2015;19(1A):A52‐A59. 10.5114/wo.2014.47129 PMC4322523 25691822

[157] Lamb MG , Rangarajan HG , Tullius BP , Lee DA . Natural killer cell therapy for hematologic malignancies: successes, challenges, and the future. Stem Cell Res Ther. 2021;12(1):211. 33766099 10.1186/s13287-021-02277-x PMC7992329

[158] Morita S , Mochizuki M , Wada K , et al. Humanized anti‐CD271 monoclonal antibody exerts an anti‐tumor effect by depleting cancer stem cells. Cancer Lett. 2019;461:144‐152. 31325530 10.1016/j.canlet.2019.07.011

[159] Choi MY , Widhopf GF 2nd , Ghia EM , et al. Phase I trial: cirmtuzumab inhibits ROR1 signaling and Stemness signatures in patients with chronic lymphocytic leukemia. Cell Stem Cell. 2018;22(6):951‐9.e3. 29859176 10.1016/j.stem.2018.05.018 PMC7001723

[160] Oriuchi N , Aoki M , Ukon N , et al. Possibility of cancer‐stem‐cell‐targeted radioimmunotherapy for acute myelogenous leukemia using (211)At‐CXCR4 monoclonal antibody. Sci Rep. 2020;10(1):6810. 32321944 10.1038/s41598-020-63557-9 PMC7176675

[161] Masuko K , Okazaki S , Satoh M , et al. Anti‐tumor effect against human cancer xenografts by a fully human monoclonal antibody to a variant 8‐epitope of CD44R1 expressed on cancer stem cells. PLoS One. 2012;7(1):e29728. 22272243 10.1371/journal.pone.0029728 PMC3260170

[162] Diessner J , Bruttel V , Stein R , et al. Targeting of preexisting and induced breast cancer stem cells with trastuzumab and trastuzumab emtansine (T‐DM1). Cell Death Dis. 2014;5(3):e1149. 24675467 10.1038/cddis.2014.115 PMC3973200

[163] Molejon MI , Tellechea JI , Moutardier V , et al. Targeting CD44 as a novel therapeutic approach for treating pancreatic cancer recurrence. Oncoscience. 2015;2(6):572‐575. 26244163 10.18632/oncoscience.172 PMC4506359

[164] Nievergall E , Ramshaw HS , Yong AS , et al. Monoclonal antibody targeting of IL‐3 receptor α with CSL362 effectively depletes CML progenitor and stem cells. Blood. 2014;123(8):1218‐1228. 24363400 10.1182/blood-2012-12-475194

[165] Richter CE , Cocco E , Bellone S , et al. High‐grade, chemotherapy‐resistant ovarian carcinomas overexpress epithelial cell adhesion molecule (EpCAM) and are highly sensitive to immunotherapy with MT201, a fully human monoclonal anti‐EpCAM antibody. Am J Obstet Gynecol. 2010;203(6):582.e1‐7. 10.1016/j.ajog.2010.07.041 PMC2993821 20870202

[166] Majeti RJO . Monoclonal antibody therapy directed against human acute myeloid leukemia stem cells. Oncogene. 2011;30(9):1009‐1019. 21076471 10.1038/onc.2010.511

[167] Marangoni E , Lecomte N , Durand L , et al. CD44 targeting reduces tumour growth and prevents post‐chemotherapy relapse of human breast cancers xenografts. Br J Cancer. 2009;100(6):918‐922. 19240712 10.1038/sj.bjc.6604953 PMC2661796

[168] Sapra P , Damelin M , DiJoseph J , et al. Long‐term tumor regression induced by an antibody–drug conjugate that targets 5T4, an oncofetal antigen expressed on tumor‐initiating cells. Mol Cancer Ther. 2013;12(1):38‐47. 23223830 10.1158/1535-7163.MCT-12-0603

[169] Herrmann I , Baeuerle PA , Friedrich M , et al. Highly efficient elimination of colorectal tumor‐initiating cells by an EpCAM/CD3‐bispecific antibody engaging human T cells. PLoS One. 2010;5(10):e13474. 20976159 10.1371/journal.pone.0013474 PMC2956687

[170] Cioffi M , Dorado J , Baeuerle PA , Heeschen C . EpCAM/CD3‐bispecific T‐cell engaging antibody MT110 eliminates primary human pancreatic cancer stem cells immunotherapy against pancreatic cancer stem cells. Clin Cancer Res. 2012;18(2):465‐474. 22096026 10.1158/1078-0432.CCR-11-1270

[171] Jin L , Lee EM , Ramshaw HS , et al. Monoclonal antibody‐mediated targeting of CD123, IL‐3 receptor α chain, eliminates human acute myeloid leukemic stem cells. Cell Stem Cell. 2009;5(1):31‐42. 19570512 10.1016/j.stem.2009.04.018

[172] Kuo S‐R , Wong L , Liu J‐S . Engineering a CD123xCD3 bispecific scFv immunofusion for the treatment of leukemia and elimination of leukemia stem cells. Protein Eng Des Sel. 2012;25(10):561‐570. 22740616 10.1093/protein/gzs040

[173] Gholamin S , Mitra SS , Richard CE , et al. Development of anti‐CD47 therapy for pediatric brain tumors. Cancer Res. 2013;73:5218.23774212

[174] Cancilla B , Cain J , Wang M , et al. Anti‐Notch1 Antibody (OMP‐52M51) impedes tumor growth and cancer stem cell frequency (CSC) in a chemo‐refractory breast cancer xenograft model with an activating Notch1 mutation and screening for activated Notch1 across multiple solid tumor types. Cancer Res. 2013;73:3728.

[175] Cao K , Pan Y , Yu L , et al. Monoclonal antibodies targeting non‐small cell lung cancer stem‐like cells by multipotent cancer stem cell monoclonal antibody library. Int J Oncol. 2017;50(2):587‐596. 28035349 10.3892/ijo.2016.3818

[176] Hart LS , Dolloff NG , Dicker DT , et al. Human colon cancer stem cells are enriched by insulin‐like growth factor‐1 and are sensitive to figitumumab. Cell Cycle. 2011;10(14):2331‐2338. 21720213 10.4161/cc.10.14.16418 PMC3322474

[177] Dallas NA , Xia L , Fan F , et al. Chemoresistant colorectal cancer cells, the cancer stem cell phenotype, and increased sensitivity to insulin‐like growth factor‐I receptor inhibition. Cancer Res. 2009;69(5):1951‐1957. 19244128 10.1158/0008-5472.CAN-08-2023 PMC3198868

[178] Mansour FA , Al‐Mazrou A , Al‐Mohanna F , Al‐Alwan M , Ghebeh H . PD‐L1 is overexpressed on breast cancer stem cells through notch3/mTOR axis. Onco Targets Ther. 2020;9(1):1729299. 10.1080/2162402X.2020.1729299 PMC7153827 32313717

[179] Zhang B , Dang J , Ba D , Wang C , Han J , Zheng F . Potential function of CTLA‐4 in the tumourigenic capacity of melanoma stem cells. Oncol Lett. 2018;16(5):6163‐6170. 30344757 10.3892/ol.2018.9354 PMC6176363

[180] Cristiani CM , Turdo A , Ventura V , et al. Accumulation of circulating CCR7(+) natural killer cells marks melanoma evolution and reveals a CCL19‐dependent metastatic pathway. Cancer Immunol Res. 2019;7(5):841‐852. 30940644 10.1158/2326-6066.CIR-18-0651

[181] Codony‐Servat J , Rosell R . Cancer stem cells and immunoresistance: clinical implications and solutions. Transl Lung Cancer Res. 2015;4(6):689. 26798578 10.3978/j.issn.2218-6751.2015.12.11 PMC4700228

[182] Meybodi SM , Farasati Far B , Pourmolaei A , et al. Immune checkpoint inhibitors promising role in cancer therapy: clinical evidence and immune‐related adverse events. Med Oncol. 2023;40(8):243. 37453930 10.1007/s12032-023-02114-6

[183] Lei MML , Lee TKW . Cancer stem cells: emerging key players in immune evasion of cancers. Front Cell Dev Biol. 2021;9:692940. 34235155 10.3389/fcell.2021.692940 PMC8257022

[184] Ai L , Chen J , Yan H , et al. Research status and outlook of PD‐1/PD‐L1 inhibitors for cancer therapy. Drug des Devel Ther. 2020;14:3625‐3649. 10.2147/DDDT.S267433 PMC7490077 32982171

[185] Du Y , Wei Y . Therapeutic potential of natural killer cells in gastric cancer. Front Immunol. 2018;9:3095. 30719024 10.3389/fimmu.2018.03095 PMC6348255

[186] Almozyan S , Colak D , Mansour F , et al. PD‐L1 promotes OCT4 and Nanog expression in breast cancer stem cells by sustaining PI3K/AKT pathway activation. Int J Cancer. 2017;141(7):1402‐1412. 28614911 10.1002/ijc.30834 PMC5575465

[187] Szaryńska M , Olejniczak A , Kobiela J , Łaski D , Śledziński Z , Kmieć Z . Cancer stem cells as targets for DC‐based immunotherapy of colorectal cancer. Sci Rep. 2018;8(1):12042. 30104575 10.1038/s41598-018-30525-3 PMC6089981

[188] Yang Y , Xu H , Huang W , et al. Targeting lung cancer stem‐like cells with TRAIL gene armed oncolytic adenovirus. J Cell Mol Med. 2015;19(5):915‐923. 25683371 10.1111/jcmm.12397 PMC4420595

[189] Chaurasiya S , Chen NG , Warner SG . Oncolytic virotherapy versus cancer stem cells: a review of approaches and mechanisms. Cancers. 2018;10(4):124. 29671772 10.3390/cancers10040124 PMC5923379

[190] Zhang Y‐N , Wang S‐B , Song S‐S , et al. Recent advances in targeting cancer stem cells using oncolytic viruses. Biotechnol Lett. 2020;42(6):865‐874. 32166558 10.1007/s10529-020-02857-6

[191] Rahman MM , McFadden GJC . Oncolytic viruses: newest frontier for cancer immunotherapy. Cancers. 2021;13(21):5452. 34771615 10.3390/cancers13215452 PMC8582515

[192] Zheng M , Huang J , Tong A , Yang H . Oncolytic viruses for cancer therapy: barriers and recent advances. Mol Ther Oncolytics. 2019;15:234‐247. 31872046 10.1016/j.omto.2019.10.007 PMC6911943

[193] Bahreyni A , Ghorbani E , Fuji H , et al. Therapeutic potency of oncolytic virotherapy‐induced cancer stem cells targeting in brain tumors, current status, and perspectives. J Cell Biochem. 2019;120(3):2766‐2773. 30321455 10.1002/jcb.27661

[194] Zhang X , Meng S , Zhang R , et al. GP73‐regulated oncolytic adenoviruses possess potent killing effect on human liver cancer stem‐like cells. Oncotarget. 2016;7(20):29346‐29358. 27121064 10.18632/oncotarget.8830 PMC5045400

[195] Jiang H , Gomez‐Manzano C , Aoki H , et al. Examination of the therapeutic potential of Delta‐24‐RGD in brain tumor stem cells: role of autophagic cell death. J Natl Cancer Inst. 2007;99(18):1410‐1414. 17848677 10.1093/jnci/djm102

[196] Marcato P , Dean CA , Giacomantonio CA , Lee PWK . Oncolytic reovirus effectively targets breast cancer stem cells. Mol Ther. 2009;17(6):972‐979. 19293772 10.1038/mt.2009.58 PMC2835173

[197] Zhu Z , Gorman MJ , McKenzie LD , et al. Zika virus has oncolytic activity against glioblastoma stem cells. J Exp Med. 2017;214(10):2843‐2857. 28874392 10.1084/jem.20171093 PMC5626408

[198] Zhu Z , Mesci P , Bernatchez JA , et al. Zika virus targets glioblastoma stem cells through a SOX2‐integrin α(v)β(5) axis. Cell Stem Cell. 2020;26(2):187‐204.e10. 31956038 10.1016/j.stem.2019.11.016 PMC9628766

[199] Hosseini M , Farassati FS , Farassati F . Targeting cancer stem cells by oncolytic viruses and nano‐mediated delivery. Onco Targets Ther. 2020;13:9349‐9350. 33061422 10.2147/OTT.S279639 PMC7519821

[200] Cintolo JA , Datta J , Mathew SJ , Czerniecki BJ . Dendritic cell‐based vaccines: barriers and opportunities. Future Oncol. 2012;8(10):1273‐1299. 23130928 10.2217/fon.12.125 PMC4260651

[201] Mastelic‐Gavillet B , Balint K , Boudousquie C , Gannon PO , Kandalaft LE . Personalized dendritic cell vaccines—recent breakthroughs and encouraging clinical results. Front Immunol. 2019;10:766. 31031762 10.3389/fimmu.2019.00766 PMC6470191

[202] Ames E , Murphy WJ . Advantages and clinical applications of natural killer cells in cancer immunotherapy. Cancer Immunol Immunother. 2014;63(1):21‐28. 23989217 10.1007/s00262-013-1469-8 PMC3880590

[203] Protzer U , Abken H . Can engineered “designer” T cells outsmart chronic hepatitis B? Hepat Res Treat. 2010;2010:901216. 21188203 10.1155/2010/901216 PMC3004001

[204] Lian G‐Y , Mak TS‐K , Yu X‐Q , Lan H‐Y . Challenges and recent advances in NK cell‐targeted immunotherapies in solid tumors. Int J Mol Sci. 2022;23(1):164. 10.3390/ijms23010164 PMC8745474 35008589

[205] Park JA , Cheung N‐KV . Limitations and opportunities for immune checkpoint inhibitors in pediatric malignancies. Cancer Treat Rev. 2017;58:22‐33. 28622628 10.1016/j.ctrv.2017.05.006 PMC5524462

[206] Sayed‐Ahmed MZ , Makeen HA , Elsherbini MM , Syed NK , Shoeib SM . Oncolytic viruses: a gene therapy for treatment of cancer in companion animals. Health Sci J. 2018;12(4):1‐9.

[207] Donini C , Rotolo R , Proment A , Aglietta M , Sangiolo D , Leuci VJC . Cellular immunotherapy targeting cancer stem cells: preclinical evidence and clinical perspective. Cells. 2021;10(3):543. 33806296 10.3390/cells10030543 PMC8001974

[208] Yao H‐J , Zhang Y‐G , Sun L , Liu Y . The effect of hyaluronic acid functionalized carbon nanotubes loaded with salinomycin on gastric cancer stem cells. Biomaterials. 2014;35(33):9208‐9223. 25115788 10.1016/j.biomaterials.2014.07.033

[209] Moaven O , Mangieri CW , Stauffer JA , Anastasiadis PZ , Borad MJ . Evolving role of oncolytic virotherapy: challenges and prospects in clinical practice. JCO Precis Oncol. 2021;5:432‐441. 10.1200/PO.20.00395 PMC8232075 34250386

[210] Ramos EK , Hoffmann AD , Gerson SL , Liu H . New opportunities and challenges to defeat cancer stem cells. Trends Cancer. 2017;3(11):780‐796. 29120754 10.1016/j.trecan.2017.08.007 PMC5958547

